# Molecular-Level Insights into Recalcitrant Ozonation Products from Effluent Organic Matter

**DOI:** 10.1021/acs.est.4c10212

**Published:** 2024-12-23

**Authors:** Elaine K. Jennings, Millaray Sierra Olea, Uwe Hübner, Rebecca Rodrigues Matos, Thorsten Reemtsma, Oliver J. Lechtenfeld

**Affiliations:** † Department Environmental Analytical Chemistry, Helmholtz Centre for Environmental Research−UFZ, Permoserstrasse 15, 04318 Leipzig, Germany; ‡ Chair of Urban Water Systems Engineering, Technical University of Munich—TUM, Am Coulombwall 3, 85748 Garching, Germany; § Institute of Analytical Chemistry, University of Leipzig, Linnéstrasse 3, 04103 Leipzig, Germany; ∥ ProVIS − Centre for Chemical Microscopy, Helmholtz Centre for Environmental Research − UFZ, Permoserstrasse 15, 04318 Leipzig, Germany

**Keywords:** advanced oxidation process, biological transformation, isotopically labeled ozone, natural organic matter, ozonation byproducts, ultrahigh resolution mass spectrometry

## Abstract

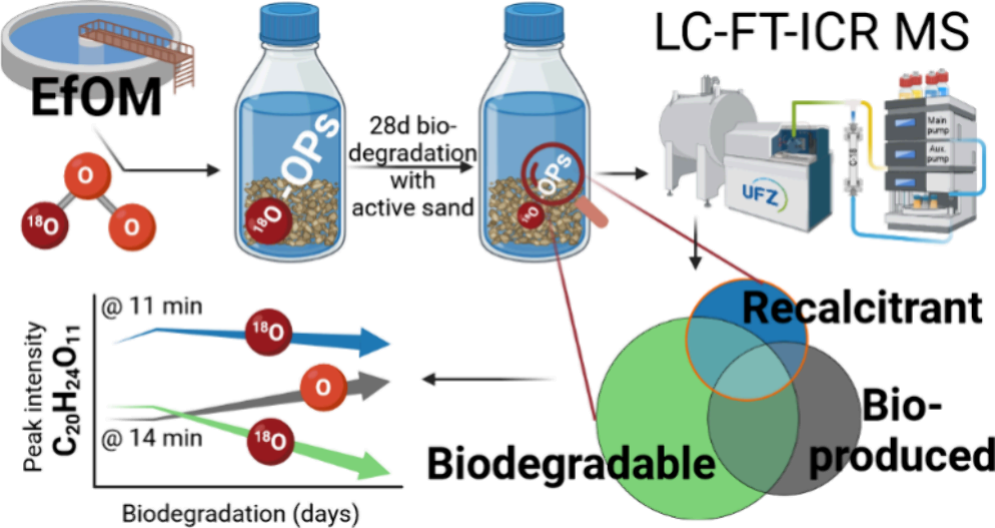

Wastewater ozonation is commonly employed to enhance the subsequent biodegradation of effluent organic matter (EfOM) and contaminants of concern. However, there is evidence suggesting the formation of recalcitrant ozonation products (OPs) from EfOM. To investigate the biodegradability of OPs we conducted batch biodegradation experiments using wastewater effluent ozonated with mass-labeled (^18^O) ozone. Molecular level analysis of EfOM was performed using reversed-phase liquid chromatography coupled with Fourier transform ion cyclotron resonance mass spectrometry (LC-FT-ICR MS) after 3 and 28 days in batch bioreactors. The use of mass labeling allowed for the unambiguous detection of OPs, with 2933 labeled OP features identified in the ozonated EfOM. Furthermore, employing polarity separation with LC facilitated the investigation of reactivity among different OP isomers. Overall, OPs exhibited a comparable proportion of recalcitrant and bioproduced molecular formulas when compared to the remaining EfOM, with 12% of OPs classified as recalcitrant and 17% bioproduced, indicating that OPs themselves are not inherently biodegradable. Additionally, recalcitrant OPs were found to be more polar based on the O/C ratios and retention time, in comparison to biodegradable OPs. Approximately one-third of the OP isomers displayed variations in their biodegradability, suggesting that studying the degradability of ozonated EfOM at the molecular formula level is heavily influenced by structural differences. Here, we offer unique insight into the biological transformation of EfOM after ozonation using labeled ozone and LC-FT-ICR MS analysis.

## Abstract 2

This work examines the biological transformation of ozonation products from wastewater effluent, showing some products remain recalcitrant to biodegradation.

## Introduction

Wastewater treatment plant effluent organic matter (EfOM) is an extremely complex mixture composed of natural organic matter (NOM), microbial products, and organic micropollutants (OMPs).^1^ Proper wastewater treatment is required to remove harmful microbes and degrade OMPs before the effluent of wastewater treatment plants (WWTP) is released into receiving water.^1−3^ One potential method for wastewater disinfection and OMP removal is the use of ozone as a chemical oxidant.^4−6^ Ozone reacts with electron-rich functional groups, breaking double bonds and adding oxygen atoms to organic molecules.^7,8^ Hydroxyl radicals produced through ozonation also react with organic molecules in a less specific reaction process. Both OMPs and EfOM are transformed during ozonation into ozonation products (OPs), but far less is known regarding OPs formed from EfOM.^9,10^ In previous work, EfOM (and other sources of NOM) has generally been shown to become more hydrophilic, oxidized, and with a lower degree of unsaturation after ozonation.^11−19^

In addition, ozonation is intended to make organic compounds more biodegradable,^20−23^ and many OMP-derived transformation products are, indeed, more biodegradable.^4,24−28^ This is especially useful in WWTP, where postozonation biological treatments are helpful in further eliminating OMPs. However, the increase in biodegradability does not appear to be generalizable and is highly dependent on the involved functional groups.^29^ In fact, some OPs (e.g., N-oxides produced from amine ozonation) have been shown to be less biodegradable than their precursors.^29−31^ Little is known about the formation of recalcitrant OPs from EfOM and the results from OMPs suggest that also EfOM-OPs will show a wide range of reactivity and thus suggest the formation of recalcitrant EfOM-OPs despite the often observed increase in assimilable organic carbon (AOC) and biological oxygen demand (BOD) after ozonation.^23,32^ As a key parameter for hazard and risk assessment, a better understanding of the biodegradability of EfOM-OPs, and the potential to form recalcitrant OPs, is critical.

In contrast to that of OMPs, where OP formation and biodegradability can be studied in isolation, the biodegradability of EfOM and OPs from EfOM is difficult to assess due to its complex chemical composition. Investigations of the biodegradability of EfOM or other DOM sources often rely on bulk organic carbon measurements, spectroscopy, or respirometry, and ozonation of organic matter has been shown to enhance overall biodegradability.^22,33−45^ However, bulk-level analysis cannot distinguish the multitudes of transformations occurring at the compound level in EfOM and few studies have investigated the biodegradability of EfOM-OPs (or other DOM) on a detailed, molecular level.^26,46−50^ Enhanced biodegradability of EfOM after ozonation is often linked to the production of small molecular-weight carbonyl compounds (as products from ozonolysis of double bonds), and it has been recently demonstrated that a large number of carbonyl-compounds are formed from EfOM and DOM after ozonation.^51,52^ However, nontargeted results from ozonation with a stable isotope-labeled O_3_ indicate that EfOM-OPs cover a wide range of masses and stoichiometric ratios.^53^ Despite generally accepted prerequisites for bioavailability of organic molecules (i.e., small and polar), trends in molecular descriptors (i.e., aggregated information from molecular formulas) with molecular-level biodegradation rates are scarce and not consistent.^54−56^ Molecular-level knowledge on the biological reactivity of EfOM-OPs in contrast to the original EfOM would thus help to link OP formation to bulk-level biodegradability.

The state-of-the-art method for molecular level investigation of EfOM and OPs in complex mixtures is ultrahigh resolution mass spectrometry, such as Fourier transform ion cyclotron resonance mass spectrometry (FT-ICR MS).^11,19,57−59^ Direct infusion (DI) is commonly used with FT-ICR MS, but cannot be used to evaluate reactivity differences of isomers since it does not incorporate any chromatographic separation.^16,60−62^ Due to measurement variability, relative net intensity changes can be unreliable, making it difficult to determine the accuracy of OP detection with conventional approaches. Some OPs remain undetectable with DI measurements, and some are potentially artifacts of data normalization and subjective intensity thresholds, leading to incomplete characterization of OPs in EfOM.^53^ Similar issues need to be considered for the study of the biodegradation of complex mixtures.

The objectives of this work are to determine (i) the biodegradability of OPs formed from EfOM and compare it to the nonozone reactive fraction of EfOM, (ii) whether molecular descriptors (e.g., molecular mass, saturation, aromaticity, polarity) can be used to explain biodegradability of OPs (and EfOM), and, in case recalcitrant OPs are formed during ozonation of EfOM, (iii) if recalcitrant EfOM-OPs have differing molecular characteristics that may impact their further fate.

To overcome previous limitations in the nontargeted analysis of EfOM, novel approaches were combined in this study to increase the reliability of OP detection and the biodegradability assessment. (1) Liquid chromatography coupled to FT-ICR MS enables the detection of different isomers based on their polarity and allows the assignment of specific reactivity (increase, decrease, or no change in intensity) both during OP formation and OP biodegradation.^15^ This improves our ability to differentiate multiple processes working on the same molecular formula, as we expect that polar OPs are formed (as compared to the remainder of EfOM) while less polar, more saturated compounds may be preferentially consumed by ozone. In addition, LC-FT-ICR MS removes the need for sample desalting and extraction boosting the detection of highly polar OPs.^15,63^ (2) Mass-labeled oxygen was also used to label OPs with heavy oxygen (^18^O).^53,64^ Evaluating the change in peak intensity of mass-labeled OPs will allow us to assign biological reactivity (degraded, produced, or recalcitrant) to OPs and separate their degradation from the remainder of EfOM as we expect only minor production of mass-labeled metabolites from mass-labeled OPs. (3) Finally, a new normalization method incorporating a labeled internal standard also improves the reliability and comparability between samples.^65^

To demonstrate the feasibility of our combined approaches, we set up a batch biodegradation experiment with municipal wastewater according to standard OECD guidelines to assess the biodegradation of EfOM and OPs, both labeled and unlabeled, on a molecular level with enhanced reliability. The results of this study will pave the way for a more detailed and reliable assessment of the effect of ozonation on EfOM degradability.

## Methods

### EfOM Collection and Experimental Design

Wastewater effluent was collected as a grab sample from the wastewater treatment plant in Garching, Germany on the morning of March 15, 2022. More details regarding the wastewater treatment plant are in the SI Section 1: Effluent Parameters. The unfiltered, nonextracted effluent was ozonated with heavy ozone following the procedures described by Sierra-Olea et al.,^64^ then added to bottles with biologically active sand as inoculum for the biodegradation experiment (SI Figure 1 and SI Table 1). Initial DOC concentration in the effluent was determined with the HTCO method (DIMATOC 2100, Dimatec Analysentechnik, Essen, Germany) in accordance with EN 1484 (TOC). All data for effluent parameters are included in the SI Section 1: Effluent Parameters. The pH, dissolved oxygen, temperature, and DOC were monitored over the course of the study and during sampling. Subsamples for DOC and LC-FT-ICR MS analysis were collected from each bottle using pre-rinsed syringe filters (Altmann Analytik Micropur CA-45/13, 0.45 um, 13 mm) initially after mixing ozonated EfOM with sand (time zero), after 3 days, and after 28 days. Hydrochloric acid (HCl, 30%, Suprapur, Merck, Darmstadt, Germany) (0.1% v/v) was added to the DOC subsamples. Subsamples for LC-FT-ICR MS were immediately capped and stored in glass vials in the dark at 4 °C before analysis. Details for the full sample list and aggregate values for each are found in SI Section 2: Sample List.

### ^18^O Ozone Generation

An ozone stock solution was generated in a modified ozonation system previously described.^64^ In short, labeled ozone was generated in a BMT 803 BT generator (BMT Messtechnik GmbH, Germany) bubbled through chilled ultrapure water for several minutes to create an ozone stock solution. Prior to ozone generation, the ozonation system was fed with ^18^O_2_ gas until approximately 50% of ^16^O_2_ was replaced and closed for recirculation of gas, generating a mixture of ^18^O/^16^O labeled ozone in the system. For accurate ozone/carbon dose, ozone stock solution concentration (46 mg/L) was determined using the indigo method.^66^ Labeled ozone was quickly mixed with the effluent to yield a final ozone-to-carbon ratio of 1 to 0.5, comparable to other studies using ozone with effluent,^67^ and a final carbon concentration of 7.6 mg/L in the ozonated effluent. The ozonated effluent was left to fully consume the ozone for 30 min prior to introduction into the batch bioreactors.

Venlafaxine was used to determine the ratio of ^18^O to ^16^O obtained from the ozone generator. A venlafaxine solution was ozonated with the ozone stock solution, and the measured ratio of venlafaxine N-oxide containing ^16^O and ^18^O was determined by positive MRM (multiple reaction monitoring) mode (here: 55%). The MRM qualifying and quantifying fragments′ *m*/*z* values were modified to include the expected labeling site and mass shift.^64^ This ratio was used to estimate the ratio of labeled to unlabeled products (i.e., isotopologues) in the EfOM samples as determined by FT-ICR MS (see Data Processing below).

### Sand Inoculation and Batch Bioreactor Set Up

Technical sand was used as a substrate for microbial community development, and this sand was placed into two large columns from a previously described system.^46^ A stepwise approach for the conditioning of the substrate was used by first using secondary effluent and subsequently ozonated secondary effluent from the Garching WWTP for a minimum of five months. The weekly ozonation of the secondary effluent was performed in a 6 L semi-batch reactor with full ozone mass balance.^68^ The ozone dose was 4 mg L^–1^ and the final volume (24 L) was transferred into a 25 L gas bag and stored at 4 °C. From this gas bag, the columns were fed by a peristaltic pump with a flow rate of 60 mL h^–1^. From these columns, the top 0.5 cm of sand (240 g) was removed and used as an inoculum for the batch biodegradation experiments. Prior to the start of the experiment, the sand was homogenized by mixing and then split in half. Half of the sand was spread into sterile Petri dishes, autoclaved (121 °C, 20 min), and then exposed to UV light (30 min) to deactivate any microbes. This autoclaved sand was split into three autoclaved amber vials to serve as an abiotic control. The rest of the sand was split into three autoclaved amber vials for the active sand reactors. The batch biodegradation experiments were performed according to Hübner et al. under oxic conditions.^69^ The ozonated effluent was added to each bottle and gently stirred to mix. Each bottle was loosely covered with foil to allow for the exchange of oxygen and placed in a dark box on an orbital shaker at 100 rpm for the duration of the experiment, 28 days.

### LC-FT-ICR MS

Since samples were not extracted and originated from the same EfOM source, samples were not diluted to the same DOC concentration to enable direct comparison of each sample at native concentrations.^63,65^ Only nonozonated effluent was diluted with ultrapure water to reach the same DOC concentration as ozonated effluent after dilution with the ozonated stock solution before direct analysis with LC-FT-ICR MS (cf. SI Table 2 for a complete list of samples).

The LC method used has been previously described.^60,63^ In short, a counter gradient was used to enhance the detection of the most polar analytes. A reversed-phase polar end-capped C18 column (ACQUITY HSS T3, 1.8 μm, 100 Å, 150 × 3 mm, Waters, Milford, U.S.A.) equipped with a guard column (ACQUITY UPLC HSS T3 VanGuard, 100 Å, 1.8 μm, 2.1 × 5 mm, Waters) was used for separation. Mobile phases were ultrapure water with 0.05% formic acid (FA) to reach pH 3 and methanol (MeOH; LC-MS-grade, Biosolve, Valkenswaard, Netherlands) to which the same amount of FA was added. In the counter gradient pump, mass-labeled internal standard naproxene-D3 was added at 50 μg/mL to both mobile phases (MeOH and ultrapure water), but without FA.^65^ Suwannee River Fulvic Acid (SRFA) from the International Humic Substances Society (SRFA II; 2S101F) and select model compounds were used for quality control of the LC system.^60^

An FT-ICR mass spectrometer equipped with a dynamically harmonized analyzer cell (solariX XR, Bruker Daltonics, Billerica, U.S.A.) and a 12 T refrigerated actively shielded superconducting magnet (Bruker Biospin, Wissembourg, France) was used for all LC-FT-ICR MS measurements in negative mode with an electrospray ionization source (Apollo II, Bruker Daltonics). More details about the FT-ICR MS settings for data acquisition have been published elsewhere.^15^ The ion accumulation time (IAT) was set to 400 ms for LC measurements to increase the signal detection of low abundance OPs, and a 4 M data size was used to enhance peak resolution. The mass range for acquisition was 147–1000 *m*/*z*. While some MFs may be present with a lower mass than those included in this range, this covers the vast majority of DOM MFs while still maintaining enough resolving power to characterize the sample effectively.

### Data Processing

Full profile LC-FT-ICR MS chromatograms were segmented into 19 one-minute wide segments between 4 and 23 min (SI Figures 2–8). These segments were then processed and treated in the same way as DI-FT-ICR MS spectra as described in previous work.^15^ Briefly, the signal-to-noise threshold was set to 4, and the spectra were internally recalibrated with a mass list of commonly found DOM masses. Mass spectral averaging and internal recalibration of segments were done in DataAnalysis 5.0 (Bruker Daltonics). After calibration, molecular formulas (MF) were assigned to peaks using in-house software with the following parameters: mass error of ±0.2 ppm, C: 1–60, H: 1–122, O: 0–40, ^18^O: 0–5, N: 0–4, S: 0–2. Other limits to formula assignments were 0.3 < H/C < 3, 0 < O/C < 1.2, 0 < N/C < 1.5, 0 < DBE (double bond equivalent) < 25, −10 < DBE-O < 10, and element probability rule.^70^ A higher O/C ratio limit was included to allow for the detection of highly oxygenated MFs.^71^

Every mass peak with an assigned MF was termed a feature (*m*/*z* × RT). If an MF was detected in LC segments, they represent different features that can be conservatively considered different isomers based on the peak width of model compounds.^60^ In this study, retention time from RP-LC is used as a proxy of molecule polarity, owing to the tight correlation between RT and NOSC.^15^

Postcolumn-infused naproxen-D3 was used to normalize the peak intensities. For every segment, the mass peak intensity of the naproxen-D3 peak (*m*/*z* 232.1058) was extracted from the spectra, and DOM/EfOM mass peak intensities were divided by the naproxen intensity to obtain an internal standard normalized peak intensity (ISN). This enhances the intersample and intrasample comparison and overcomes the limitations of other typically used normalization methods for OM analysis with LC.^15,65^ More details regarding this normalization method and a comparison between it and commonly used normalization methods (i.e., the sum of intensity and base peak) are included in SI Section 4: Internal Standard Normalization (SI Figures 9 and 10).

To limit false assignments due to the inclusion of ^18^O during formula assignment, MF were filtered in a workflow previously described for mass labeled data (SI Figure 11).^53^ In short, validation of ^18^O formulas required detection in at least two of three samples in the same treatment group, a continuous series of isotopologues detected, and an intensity ratio of ^16^O and ^18^O peaks of less than 10.3 (see SI Section 5: 18O/16O Data Filtering for calculation, SI Tables 3, 4, SI Figures 12 and 13), and the mass difference between the expected and measured mass of the ^18^O isotope from the ^16^O isotopologue was limited to 2.00424 ± 0.00015 Da. In addition, all formulas found in blanks were subtracted from the samples, and formulas with ^18^O found in both nonozonated and ozonated EfOM samples were also removed. The remaining ^18^O-containing MF were defined as *labeled ozonation product* (OP).

*Unlabeled OP*s were defined as MFs with an increase in ISN between the nonozonated and ozonated sample or which were only detected after ozonation. MFs that did not show a (net) ISN change or decreased in their ISN upon ozonation were classified as *nonreactive* and *depleted*, respectively.

At the end of this data filtering, a list of all formulas and OPs detected after ozonation was created, and these MFs were tracked during the experiment to evaluate persistence and biodegradability. This process was repeated for each LC segment, resulting in retention time-specific biodegradation data for each MF, including labeled OPs.

Biodegradability was determined based on calculating the percent ISN change (δISN) of each peak over time. The average ISN for each MF was calculated from the ISN obtained from three batch bioreactors. δISN for each MF was then calculated by subtracting the average normalized intensity of each peak after 3 and 28 days from the average normalized intensity at time zero and dividing by the average normalized intensity at time zero (eq 1).
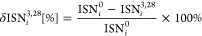
1

If the ISN of an MF *i* decreased more than 30% after 3 days (δISN^3^ ≤ −30%), the MF was classified as *readily biodegradable*. If the MF decreased in average intensity by more than 30% after 28 days (δISN^28^ ≤ −30%), but not after 3 days (δISN^3^ > −30%), the MF was classified as *biodegradable*. If the percent intensity change was between 30 and −30% after 28 days (−30% ≤ δISN^28^ ≤ 30%), the MF was classified as *recalcitrant*. All MFs with an intensity increase of more than 30% after 28 days (δISN^28^ > 30%), were classified as *bioproduced*. The 30% threshold was chosen as a more relaxed threshold compared to the 60% threshold used in standard OECD biodegradability tests, since in this study we used individual MF intensities, not bulk-level analysis like DOC to assess biodegradation.^72^ Finally, all MFs not detected or found in only one out of the three bottle replicates after 3 or 28 days were classified as *fully removed*. For details about the classification cf. SI Section 7: Reactivity with Ozone and Biological Treatment. All MFs with higher removal (or enrichment) in the abiotic controls compared to the active sand bioreactors were not considered for further analysis. Molecular descriptors between classes were compared with a two-sample *t-*test, and α = 0.05.

### Modeling of Biodegradation

Correlations between molecular descriptors and biodegradability were calculated both for single variables using Pearson correlation and in a multivariable linear model using R (version 4.0.4). All variables used (H/C, NOSC, DBE-O, retention time, and *m*/*z*) were first standardized for the multivariable model to eliminate issues related to the differing variable scales. Standardization was done by calculating the mean of each variable, setting the mean to zero, and rescaling the standard deviation to one. O/C was not considered in the multivariable linear model due to high collinearity (variance inflation factor over 5) with other molecular descriptors included in the model. A significance level of α = 0.05 was used for univariate and multivariate models. A random selection of 80% of all EfOM MFs which were not classified as *bioproduced* in the sample was used to build the model, and the remaining 20% of the data set was used to test the model for predictive capacity. More details regarding both the univariate and the multivariable model can be found in SI Section 8: Correlation between the Molecular Descriptors and Biodegradability.

## Results and Discussion

### Biodegradability of Ozonated Effluent Organic Matter

The biodegradation of EfOM ozonated with labeled ozone was followed over 28 days by sampling after 3 days and 28 days. In the active sand batch bioreactors, dissolved organic carbon decreased on average by 38% after 3 days and increased again by 41% after 28 days (overall net decrease: 13%; see SI Table 5 and SI Figure 14). This change of bulk DOC concentration is similar to previous work investigating the bulk level biological transformation of ozonated DOM, found to be between 17 and 48% when using biologically active sand carbon filters after ozonation,^37^ and between 34 and 46% when ozone was coupled to slow sand filtration.^73^ In contrast, the autoclaved bottles, although starting at a much higher initial DOC concentration, decreased by 20% after 3 days (likely due to sorption effects) and remained constant after 28 days.

In the ozonated EfOM, 4,144 unique MFs were detected by LC-FT-ICR MS and evaluated for biodegradability (Table 1). On average, MFs were detected in 3.4 segments over the course of the chromatographic run, although most MF (*n* = 1,849) were detected in only one segment (SI Figure 24). When accounting for multiple detections, over 14,100 features were detected in ozonated EfOM (Table 1). Out of those, 6,316 features (45%) could be classified as OPs based on intensity changes (*n* = 3,383) or the presence of ^18^O labeling (*n* = 2,933; Table 1). Interestingly, most CHO features are classified as either *depleted* (39%) or as *ozonation products* (44%) during ozonation, with only 17% remaining *unchanged* after ozonation. This shows that most CHO formulas are more reactive with ozone compared to the other formula classes that show a higher abundance of unchanged MFs (see SI Tables 6 and 7). Notably, EfOM reactivity toward ozone is complex and depends on many factors like sample type and ozone dose.^16,52,74^

**Table 1:** Number of Features and Unique Molecular Formulas (MF) Detected in EfOM after Ozonation (Data Shown for Sample #2, cf. SI Table 2), Classified Based On the Reactivity with Ozone^a^

	number of features	number of unique MF	sum normalized peak intensities	average normalized peak intensity (10^–3^)
*labeled* OP^b^	2,933	655	13.0	4.42
*unlabeled* OP^b^	3,383	2,294	36.4	10.8
*depleted* EfOM^c^	4,817	2,530	10.9	2.26
*nonreactive* EfOM^d^	2,970	2,047	9.61	3.24
total EfOM	14,103	4,144	69.9	4.96

a The data include features and MF that were subsequently removed from the biodegradation evaluation due to their behavior in the abiotic control (*n* = 2,491). Sum and average peak intensity of all features calculated based on the post-column internal standard normalization

b *Labeled* ozonation products (OPs) are features/MF that carry an ^18^O after ozonation, while *unlabeled* OPs were identified based on an increase in intensity after ozonation (cf. Methods section).

c *Depleted* features/MF show a lower intensity after ozonation and represent the fraction of EfOM that reacts with ozone.

d *Nonreactive* features/MF do not show a net change in intensity after ozonation and represent the fraction of EfOM that is not reactive under the experimental conditions.

**Figure 1:**
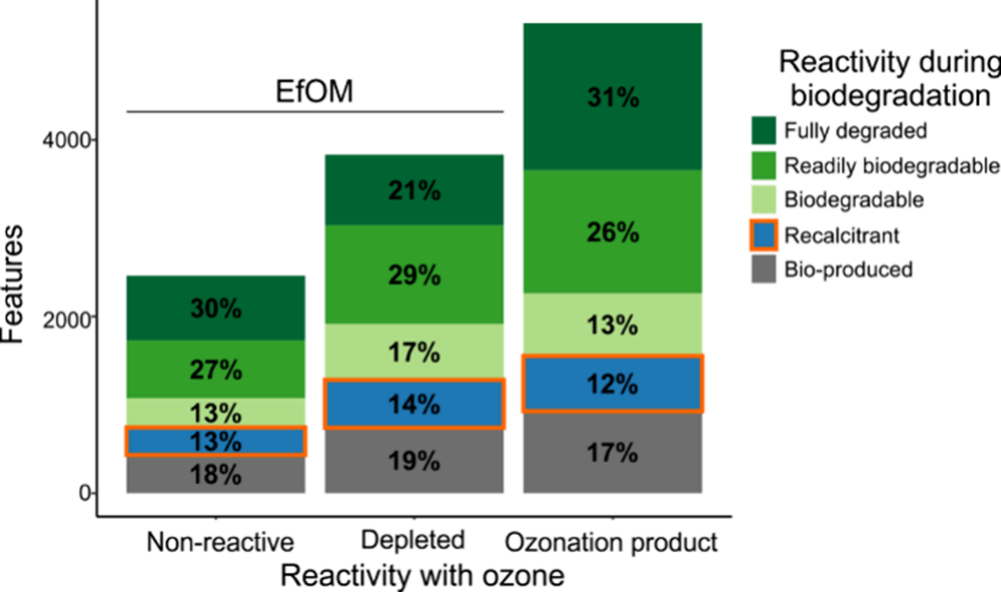
Number of features in EfOM after biodegradation classified according to their reactivity toward ozone (*nonreactive*, *depleted*, *ozonation products*, OPs). The MF are further classified according to their biodegradability (colors) and the relative number of MF within each ozone reactivity class is displayed as the percent value. Note that nonreactive and depleted features represent the original EfOM present before ozonation and that the OPs contain ^18^O-labeled and unlabeled OPs.

Biodegradability was assessed for each feature based on the percent intensity changes (δISN) after 3 and after 28 days and classified accordingly (SI Tables 8 and 9). 67–70% of all EfOM features were susceptible to biodegradation, regardless of whether they are part of the original EfOM (i.e., *depleted* and *nonreactive*) or produced during ozonation (Figure 1). Unexpectedly, 29% of OPs are either *recalcitrant* or *bioproduced*, similar to the *depleted* and *nonreactive* EfOM (33 and 31%, respectively). *Nonreactive* EfOM and OP precursors (i.e., *depleted* after ozonation) displayed an overall similar biodegradability pattern despite different total number of features (Figure 1). Together, both classes reflect the bulk EfOM biodegradation potential prior to ozonation. Our results demonstrate that recalcitrant OPs are produced during ozonation and that they represent a substantial fraction of all OPs, comparable with recalcitrant compounds being present before ozonation. Of note, more OPs (labeled and unlabeled) are *fully removed* after biodegradation as compared to *depleted* EfOM (31% vs 21%), owing at least partially to their on average lower intensity (Figure 1 and Table 1).

While it is well established that ozonation increases the biodegradability of many OMPs and of EfOM at the bulk concentration level,^30^ the comparable reactivity between EfOM-OPs and the remainder of compounds in EfOM shows that OPs cannot be considered per se as a driver for the increase in biodegradability of ozonated EfOM. Our study confirms that ozone does produce OPs which are (readily) biodegradable, but the large fraction of recalcitrant and bioproduced OPs found here implies that enhanced biodegradability cannot be assumed for all OPs, reflecting observations from OMPs. We note that in contrast to OMP studies the relationship between the biodegradability of a precursor and its OP cannot be directly established for complex EfOM. In order to account for the commonly observed higher biodegradation of ozonated EfOM on the bulk level,^23,32^ quantitatively more OPs must be produced from initially recalcitrant EfOM as compared to (already) biodegradable EfOM. In order to better understand the determinants for biodegradable and recalcitrant OPs, the relationship between biodegradability and molecular descriptors/polarity will be investigated in the following.

### Molecular Characteristics of Recalcitrant and Biodegradable Ozonation Products

**Figure 2:**
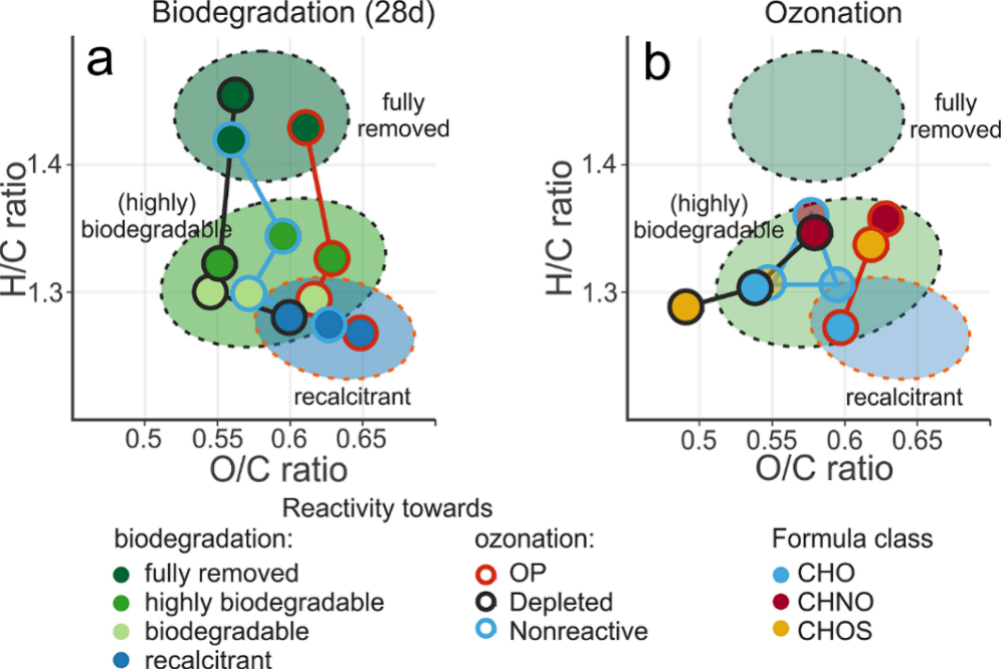
Number-averaged molecular descriptors of EfOM features according to their reactivity classification upon (a) biodegradation (color of circle filling) and (b) ozonation (color of circle border). The *bioproduced* class (i.e., features that had a higher intensity after biodegradation) and error bars were omitted for clarity. Colored ellipses are shown to indicate biodegradability classes from panel (a) in the ozone reactivity panel (b). Note that the classes in b are split based on formula class, and OPs contain ^18^O-labeled and unlabeled OPs. The lines in panels a and b are visual aids connecting the same ozone reactivity class.

The *biodegradable* and *highly biodegradable* features were very similar based on aggregated molecular descriptors, with *highly biodegradable* features being slightly more saturated (higher H/C) and of smaller mass (data not shown) as compared to the *biodegradable* features (Figure 2a). Both classes will thus be subsumed under a “*biodegradable*” category in the following sections. The *fully removed* fraction was characterized by the highest H/C ratios and largest mass, regardless of their ozone reactivity classification. In contrast, *recalcitrant* features represent the most oxygenated (high O/C) and unsaturated (low H/C) molecules.

At first inspection, OPs fall on average in the same range of molecular H/C and O/C ratios as the *biodegradable* fraction, indicating that biodegradable OPs may be formed from EfOM present before ozonation. However, also features *depleted* after ozonation and *nonreactive* features share a large overlap with the *biodegradable* class (Figure 2b). Together with the quantitative data from Figure 1, this suggests that the production of *biodegradable* OPs is counterbalanced by the fact that also biodegradable EfOM precursors react with ozone and are not available for further biodegradation. Likewise, also the *recalcitrant* OPs are not readily distinguishable from *recalcitrant* molecules present before ozonation (*depleted* and *nonreactive)*, based on molecular descriptors (Figure 2a and SI Figure 15). This can further explain why also a substantial fraction of recalcitrant features were produced from ozonation: The molecular-level changes upon ozonation may not be sufficient to render a recalcitrant EfOM molecule bioavailable. In fact, also many OMPs can form stable OPs, depending on their reactive sites and functional groups.^29,75^

### Molecular-Level Drivers of Biodegradability

**Figure 3:**
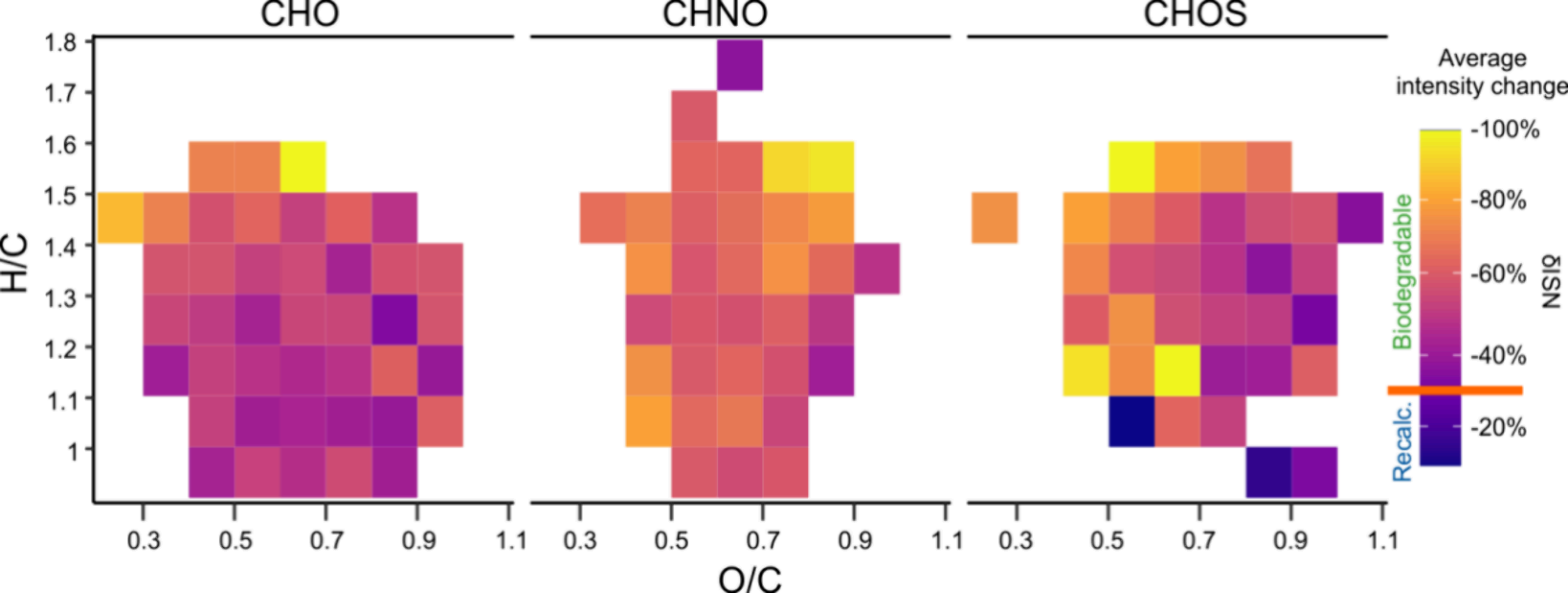
Aggregated average peak intensity change (δISN, colors) of ^18^O-labeled OPs after 28 days of biodegradation, based on their O/C and H/C values and separated by formula class. δISN values below −30% (orange line in the color bar) indicate biodegradable OPs. Each tile represents ≥3 ^18^O-labeled OPs (mean = 16). The *produced* class was omitted.

Biodegradability differed among formula classes. CHNO formulas were characterized by overall higher removal (average of −63% δISN for all OPs of the *biodegradable* and *fully removed* class), while CHOS OPs had on average a lower removal during the 28 days experiment time (average of −51% δISN) compared to CHO (−56% δISN, Figure 3). The higher removal of CHNO MFs may be partially explained by the preferential microbial consumption of nitrogen-containing EfOM.^48,76^ Many CHOS features with high H/C and low O/C ratios that may be attributed to omnipresent surfactants in EfOM show higher removal compared to other CHOS compounds.^11,46^ However, many CHOS formulas remain recalcitrant during this study, especially with low saturation and higher oxygen content supporting previous work showing higher recalcitrance among CHOS formulas found in landfill leachates and EfOM.^46,48^

CHO formulas, although substantially contributing to the overall ozone reactivity and new OPs (SI Tables 6 and 7), were the least reactive with respect to biodegradation (Figure 3). Notably, ozonation increases biodegradability only for the CHO molecules (*nonreactive* and *depleted*: −53% δISN), while CHNO and CHOS formulas showed a similar degree of degradation in bulk EfOM. Molecules composed only of CHO represent a DOM background in EfOM and are devoid of any heteroatomic functional group that is reactive with ozone (e.g., amines, sulfides, etc.). Structural changes during ozonation of CHO compounds in EfOM can be related to the breaking of double bonds or the opening of aromatic rings. The consistently lower DBE-O and higher NOSC values (indicating fewer double bonds and/or more oxygen atoms) of *recalcitrant* as compared to *biodegradable* OPs (*p* < 0.05, SI Figure 16) suggest that structural changes during ozonation cannot be easily linked to biodegradability. Here, the multiple pathways being active during ozonation (direct O-transfer, OH-radical) may easily lead to an immense spectrum of potential OP structures.^77^

Further investigation at the individual molecular formula level revealed a significant positive correlation (*p* < 0.05) with biodegradation for H/C, NOSC, DBE-O, retention time, and *m*/*z* both in individual linear models and multiple variable linear models with H/C having the strongest influence on biodegradability (SI Table 10). When separated at the formula class level, the correlation between biodegradability and H/C ratio was significant for CHNO and CHOS formulas, but not for CHO formulas (SI Figure 17). In contrast, biodegradability correlated significantly negatively with NOSC and O/C and positively with DBE-O for CHO formulas (*p* < 0.05 for all; SI Figures 18–21). In this study, molecular mass appeared to have only a weak effect on biodegradability (SI Table 10 and SI Figure 21).

The alignment of the biodegradation reactivity with molecular H/C ratios was previously observed in other batch biodegradation experiments and related to the rapid heterotrophic utilization of aliphatic DOM.^55,78^ However, also molecule polarity (based on O/C, NOSC, or reversed-phase LC retention) is frequently discussed as a main driver for bioavailability and degradability,^54,55^ aligning with the results from this study (SI Figure 11 and SI Table 10). Despite the significance of the individual correlations, the multivariable model indicates that the molecular descriptors used only explain about 6% of the removal in this 28-day biodegradation experiment at the level of molecular formulas, with overall low predictive power (SI Figures 22 and 23). Overall, the substantial overlap between aggregated molecular descriptors of *biodegradable* and *recalcitrant* EfOM as well as the large range of observed removal in our study suggests that the molecular composition and stoichiometry may not be the main driver for the biodegradation and warrants a closer inspection of the biodegradability at the level of individual isomers.

### Isomer-Resolved Biodegradability

**Figure 4:**
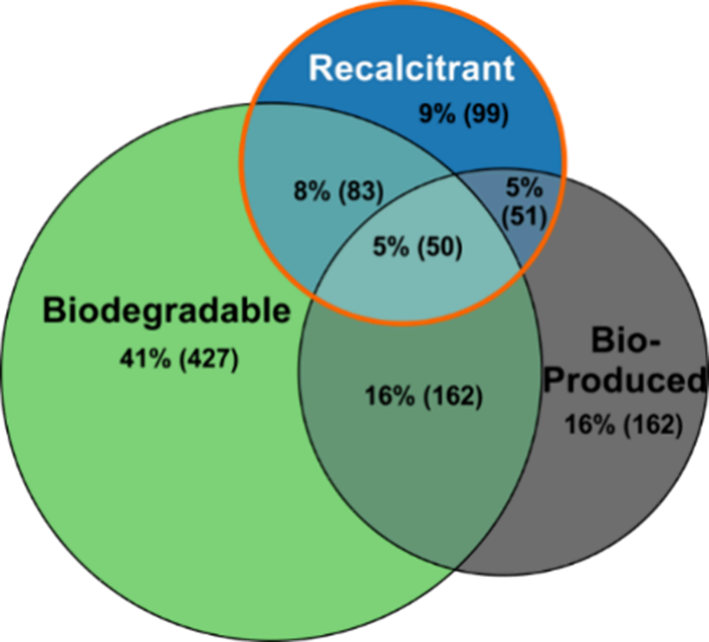
Proportions of OPs with ^18^O_1_ according to their behavior in the biodegradation experiment. If all segments in which an OP molecular formula was found had a consistent percent intensity change, this OP count toward *biodegradable* (−30% < δISN), *recalcitrant* (−30% < δISN < 30%), or *bioproduced* (δISN > 30%). Isomers of the same MF, which differ in their behavior depending on the retention time of elution, are located in the intersections. Note that the *biodegradable* and *readily biodegradable* class are combined.

By coupling LC to FT-ICR MS, we can observe how isomers of OPs (based on their occurrence in different one-minute segments) differ in their biodegradability. 34% of all ^18^O-labeled OPs had differing reactivity depending on retention time and 50 labeled OPs (5%) were classified as having all three major reactivity classes (Figure 4, SI Figure 25 and SI Table 11). When considering all features in EfOM, most MF were detected in more than one segment, and only 14% of these putative isomers had the same removal classification at different retention times.

**Figure 5:**
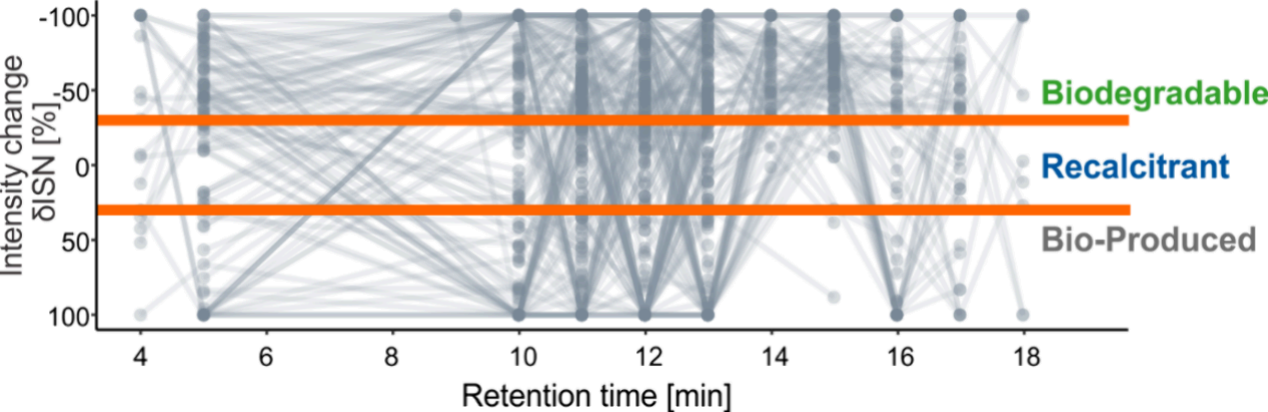
Change in biodegradability classification over the retention time for ^18^O-labeled OPs detected over more than one retention time. Lines connect each molecular formula (MF) across retention time, connecting the isomers. The intensity change δISN was limited to 100% (i.e., doubled intensity after biodegradation) and set to −100% for fully removed formulas for visualization purposes. MF below the blue line classified as *bioproduced* (δISN > 30%), MF above the green line as *biodegradable* (−30% < δISN, including *readily biodegradable* and *fully removed* MF), and MF in the middle as *recalcitrant* (−30% < δISN < 30%). The line color intensity corresponds to the number of overlapping MFs. Note that the *y*-axis is reversed.

Pronounced production of polar OPs (with lower retention time) has been previously observed with LC-FT-ICR MS, while less polar isomers of the same MF were consistently depleted during ozonation.^15^ In contrast, no consistent relationship existed between biodegradability and retention time (Figure 5). While *recalcitrant* OPs tend to have on average a lower retention time than other OPs (SI Figure 11), this trend does not necessarily apply to isomers found in consecutive segments (Figure 5). This highlights the benefits of using LC-FT-ICR MS, which can detect subtle changes at the isomer level while conventional DI-FT-ICR MS can only reveal isomer-aggregated changes thus masking the large reactivity differences at the level of EfOM isomers.^15^ The large variability of biodegradation at the isomer level questions a simple relationship between molecular composition and biodegradability and might explain the low explanatory power of molecular descriptors (SI Figure 11 and SI Table 11) and the inconsistent observations regarding DOM degradability across ecosystems.

**Figure 6:**
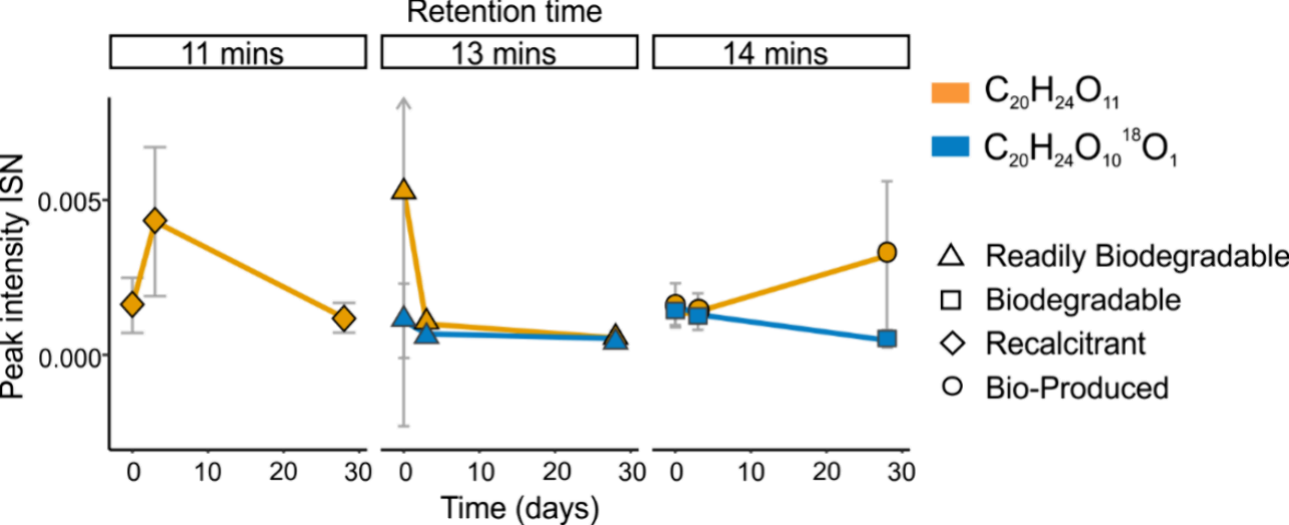
Peak intensity changes during biodegradation (sampling at 0, 3, and 28 days) for one selected OP (C_20_H_24_O_11_, orange line and symbols) and its ^18^O-labeled form (C_20_H_24_O_10_^18^O_1_, light blue line and symbols) at different retention times. Error bars indicate the variability between the triplicate bioreactors used during this experiment and not the instrument variability. Note that at 14 min, both isotopologues differ in their reactivity.

An example of isomeric differences in biodegradability shows one OP (C_20_H_24_O_11_) classified as recalcitrant at 11 min, readily biodegradable at 13 min, and bioproduced at 14 min (Figure 6).

Notably, ^18^O-labeled OPs can have different biodegradability classifications than their unlabeled isotopologues even at the same retention time (Figure 6). This applies to 199 of 1309 features with one ^18^O addition and 47 of 107 features with ^18^O_2_. For the example OP C_20_H_24_O_10_^18^O_1_ in Figure 6 at 14 min, the ^18^O isotopologue is classified as biodegradable while the ^16^O isotopologue C_20_H_24_O_11_ is classified as bioproduced after 28 days. This implies that the ^16^O isotopologue is formed during the biological process, while the ^18^O-containing feature, being unequivocally identified as OP, is degraded. With the ^18^O label acting as a clear marker of OP production, ^18^O-labeling can also be used to reliably track the degradation of OPs. The ^16^O isotopologues, in contrast, mostly represent a mixture of OPs (formed together with the ^18^O-labeled OPs), endogenous compounds, and newly produced compounds from biological activity. It appears likely that the differing reactivity (of a feature with the same *m*/*z* and same RT, but different isotopologues) is related to different structures between the OP and the endogenous/bioproduced compounds. Labeled ozone thus also improves our ability to study the biological transformation of EfOM by limiting the number of interfering isomers so that different processes can be studied concurrently.

Overall, our results imply that the majority of isomers in EfOM and its OPs have varying levels of biodegradability and that an assessment of individual isomers is needed to better understand the biodegradation of EfOM and OM in general. This will require a combination of efficient separation techniques, (isotope) labeling approaches,^52,53^ further structural information via tandem MS,^79,80^ as well as novel machine learning-based data evaluation.^81,82^

### Limitations of the Approach and Future Research Directions

With the combination of LC-FT-ICR MS and stable isotope labeling, recalcitrant OPs can be detected in more detail than previously studied, showing both isomeric and isotopologue differences in reactivity. The incorporation of labeled ^18^O aids in the determination of the transformation of OPs, both during the ozonation process as well as during the biological treatment of EfOM. In this study, we did not distinguish between the various ozone reaction mechanisms leading to direct O-transfer or OH-radical-induced transformation.^53^ The production of recalcitrant OPs implies that other oxidative treatment processes (e.g., OH radicals) may also produce recalcitrant compounds.

Increased polarity of OPs coupled with decreased biodegradability means the recalcitrant OPs could be both more mobile as well as more persistent in the aquatic environment compared to other OPs representing a potential source of currently unregulated persistent and mobile (PM) substances.^83^ Further work to investigate the potential effects of these highly polar and recalcitrant OPs is needed to better understand any potential environmental risk.

Enhanced biodegradation of ozonated EfOM has been linked to the production of small molecular weight carbonyl compounds.^32,51^ Here, we find evidence that ozonation produces a mixture of biodegradable and recalcitrant OPs from EfOM. In particular, depleted EfOM-MF, the presumed precursors of OPs, shows a similar fraction of recalcitrant MFs as compared to OPs. Further, ozonation produces OPs with similar molecular descriptors as recalcitrant EfOM. This indicates that OP themselves are not necessarily more biodegradable than other components of EfOM and that the biodegradation enhancement may depend on the initial fraction of degradable and recalcitrant EfOM. However, this requires further investigation of more samples, also from various WWTPs and seasons and different ozone doses, to confirm. Also, quantitative links between changes in biodegradable and recalcitrant fractions and measured AOC/BOD values have yet to be established.

The variable biodegradation at the level of individual isomers and the weak relationships to molecular descriptors emphasize that more information at the structural level (e.g., via linkage to carbonyl functionalities)^52^ is necessary to explain the observed biodegradation pattern of OPs.^84^ However, direct links between precursors and their OPs cannot yet be established for complex EfOM, despite the use of ^18^O-labeled ozone. This would be required to better link transformations and changes in biodegradability at the individual compound level.

Finally, our results also shed light on a long-lasting question that revolves around the reasons for the biodegradability and recalcitrance of OM in general,^85,86^ and underpins that simple molecular descriptors may be insufficient to capture the complex interplay between community structure, metabolic potential, and environmental factors as relevant factors for organic matter turnover.

## Data Availability

Processed and quality checked data for all samples and segments and final list of 14,103 molecular formulas with ozonation and biodegradation classification are available from the UFZ Data Investigation Portal: https://doi.org/10.48758/ufz.15360. Raw MS files can be shared upon request.

## Supporting Information Available

Additional details about the collected wastewater treatment plant effluent; detailed sample list and processing schema; compilation of total ion chromatograms of all samples; description of the internal standard normalization method; ^18^O adjusted FT-ICR MS data filtration schema; DOC and pH values; further details about the EfOM reactivity during ozonation and biodegradation treatment; details about the biodegradation modeling results; and description of the isomer distribution (PDF)

## Notes

Table of contents graphic created in BioRender. Lechtenfeld, O. (2024) https://BioRender.com/w21r519.
