# Supplementary material for: Molecular-Level Insights into Recalcitrant Ozonation Products from Effluent Organic Matter
Source: Environ Sci Technol. 2024 Dec 23;59(1):823–33. doi: 10.1021/acs.est.4c10212 (PMC11741107; doi:10.1021/acs.est.4c10212)
Supplement: Supplementary file 1 — es4c10212_si_001.pdf [file es4c10212_si_001.pdf]

Supporting Information for

Molecular Level Insights into Recalcitrant Ozonation Products from Effluent Organic Matter

*Elaine K. Jennings[a], Millaray Sierra Olea[b], Uwe Hübner[b], Rebecca Rodrigues Matos[a], Thorsten Reemtsma[a,c], and Oliver J. Lechtenfeld[a,d]**

AUTHOR ADDRESS

a. Department Environmental Analytical Chemistry, Helmholtz Centre for Environmental Research–UFZ, Permoserstrasse 15, 04318 Leipzig, Germany.

b. Chair of Urban Water Systems Engineering, Technical University of Munich—TUM, Am Coulombwall 3, 85748 Garching, Germany.

c. Institute of Analytical Chemistry, University of Leipzig, Linnéstrasse 3, 04103 Leipzig, Germany

d. ProVIS–Centre for Chemical Microscopy, Helmholtz Centre for Environmental Research–UFZ, Permoserstrasse 15, 04318 Leipzig, Germany.

* Corresponding author: oliver.lechtenfeld@ufz.de

This file contains

25 figures

11 tables

on 28 pages

Contents

1. Effluent Parameters 5

2. Sample List 5

SI Figure 1: Visual Scheme of all samples included in the study. In the sample description, t0 means samples taken at the start of the experiment, t3 means samples taken after 3 days, and t28 means samples taken after 28 days. 5

SI Table 1: Set up of active and autoclaved sand bottles. 6

SI Table 2: List of all samples measured with LC-FT-ICR MS and the aggregate values of each sample. All assigned peaks include multiple assignments (only counted once). Intensity Sum includes all the assigned peak intensities added up for all segments in the chromatogram. In the sample description, t0 means samples taken at the start of the experiment, t3 means samples taken after 3 days, and t28 means samples taken after 28 days. 6

3. Total Ion Chromatograms 7

SI Figure 2: Ozonated (red) and non-ozonated (blue) EfOM, not mixed with activated sand. 7

SI Figure 3: Activated sand bottles 1 (purple), 2 (blue), and 3 (green) immediately after mixing (time=0). 7

SI Figure 4: Activated sand bottles 1 (purple), 2 (blue), and 3 (green) after 3 days of shaking in the dark (t=3). 7

SI Figure 5: Activated sand bottles 1 (purple), 2 (blue), and 3 (green) after 28 days of shaking in the dark (t=28). 8

SI Figure 6: Autoclaved (inactive) sand bottles 1 (red), 2 (orange), and 3 (yellow) immediately after mixing (time=0). 8

SI Figure 7: Autoclaved (inactive) sand bottles 1 (red), 2 (orange), and 3 (yellow) after 3 days of shaking in the dark (t=3). 8

SI Figure 8: Autoclaved (inactive) sand bottles 1 (red), 2 (orange), and 3 (yellow) after 28 days of shaking in the dark (t=28). 9

4. Internal Standard Normalization 10

SI Figure 9: Repeatability of the MCs expressed as the coefficient of variation (CV) of mass peak intensity across triplicate injections. MCs were spiked in different SRFA carbon concentrations: 2.0, 2.5, 5.0, 10 and 15 mg/L C. The CVs were calculated for total ion count (SUM, blue), internal standard (ISN, green), and base peak (BPK, pink) normalization. 11

SI Figure 10: Distribution of Pearson's correlation coefficient for individual DOM MFs, grouped and colored by normalization method (across concentration range 2 – 15 mg L-1). The correlations were calculated independently for each segment (RT 11 - 18 min) and then combined. 12

5. $^{18}O/^{16}O$ Data Filtering 13

SI Table 3: Expected Ratios of $^{18}O/^{16}O$ with 55% abundance of ^{18}O 13

SI Table 4: The limit of $^{18}O/^{16}O$ ratios, with 65% ^{18}O abundance maximum 13

SI Figure 11: Data processing workflow outline. 14

SI Figure 12: After filtering, the number of ^{18}O isotopes added to all OPs detected. 1414 OPs were detected with one ^{18}O label while 107 were detected with two. Dark blue represents features without any label, while lighter blue represents formulas with the ^{18}O present. 14

SI Figure 13: The O/C ratio of all features detected in ozonated EfOM with LC-FT-ICR MS. All OPs are in orange, and the rest of EfOM is in green. 15

6. Bulk parameter 16

SI Table 5: Change in pH, dissolved oxygen, and DOC concentration in bottles over time. 16

SI Figure 14: Change in DOC concentration over time in each bottle. Blue: activated sand, green: autoclaved sand. 16

7. Reactivity with Ozone and Biological Treatment 17

SI Table 6: Formula class distribution based on reactivity with ozone. 17

SI Table 7: Distribution of formula classes in EfOM MF with different reactivity towards ozone. 17

SI Table 8: Thresholds for biodegradability classification 18

SI Table 9: Total number of features found in ozonated EfOM based on removal classification (abiotic removed not included) and the average retention time for each removal class. 18

SI Figure 15: Molecular H/C vs O/C (a) and DBE-O vs O/C (b) values of FT-ICR MS derived molecular formulas and their reactivity classification with ozone (ozonation products, OP, depleted and non-reactive EfOM). Only CHO-containing MF are shown.. 19

*SI Figure 16: Comparison of biodegradable OPs (including readily biodegradable and biodegradable in one group) (green, n = 1015) and recalcitrant OPs (blue, n = 317) by their molecular descriptors NOSC, O/C, DBE-O, and their retention time in LC-FT-ICR MS, showing only labeled OPs. Half violins are scaled based on the number of detected MFs in each group. The significance levels of differences in mean values is indicated with ***: p-value < 0.001 and ****: p-value < 0.0001.* 20

8. Correlation between Molecular Descriptors and Biodegradability 21

SI Table 10: Multivariable linear model coefficients correlating biodegradability with H/C, NOSC, DBE-O, retention time, and m/z for all recalcitrant, biodegradable, readily biodegradable, and fully removed MFs. The R^2 of the model is 0.064, residual standard error is 0.97, and root mean squared error is 1.0, all based on standardized variables. 21

SI Figure 17: Linear correlation between percent removal and H/C ratio. 22

SI Figure 18: Linear correlation between percent removal and O/C ratio. 22

SI Figure 19: Linear correlation between percent removal and formula NOSC. 23

SI Figure 20: Linear correlation between percent removal and formula DBE-O. 23

SI Figure 21: Linear correlation between percent removal and molecular weight. 24

SI Figure 22: The fit of the predicted removal and actual removal of 20% of the EfOM data based on the multivariable linear model. The red dashed line indicates where the model perfectly predicts the

removal, while dots below represent cases where the model's predictions underestimate the actual removal (red) and dots above are overestimated by the model (blue). 25

SI Figure 23: Residual plot from the multivariable linear model. Since the data are not evenly distributed around 0, the model does not predict the data well. 25

9. Isomers in LC-FT-ICR MS 26

SI Figure 24: The number of multiple detections over the chromatographic separation for all EfOM molecular formulas. Each detection represents at least one isomer present in the sample. 26

SI Table 11: How many MFs with isomers are detected with different removal classifications (readily biodegradable, biodegradable, recalcitrant, bio-produced, or fully removed). 27

SI Figure 25: The number of different removal classifications assigned to each MF and the number of times it was detected in LC-FT-ICR MS. The intensity of the colored dots represent the number of MFs with this many detections and removal classifications, with the lightest dots (white) equal to one and the darkest dot (two detections and two different classifications) equal to 698 MFs. The maximum number of distinct classification = 5, relating to the different removal classifications included (readily biodegradable, biodegradable, recalcitrant, bio-produced, or fully removed). 27

10. Additional References 28

1. Effluent Parameters

Effluent was collected from the wastewater treatment plant in Garching, Germany (WWTP, 31,000 PE). This WWTP treats the municipal wastewater from the city as well as the University and research campus. It employs a combination of activated sludge and trickling filters for biological nutrient removal. Additional UV disinfection is applied during summer months (May-October) to ensure bathing water quality in the receiving river Isar. Effluent samples were collected in March 2022 during operation without UV disinfection.

The following parameters were recorded for the effluent used for this study. Small volumes (2 mL) were taken during subsampling for DOC and LC-FT-ICR MS, leaving the majority of the volume inside each bottle. The temperature remained stable at 20°C over the course of the experiment. DOC concentration: initial EfOM DOC concentration 11.28 mg/L, and 7.6 mg/L after ozonation (due to dilution of the sample, not mineralization of the carbon). Autoclaved sand initially has much higher DOC due to the destruction of microbes during the autoclaving process, which releases DOC into the system.

2. Sample List

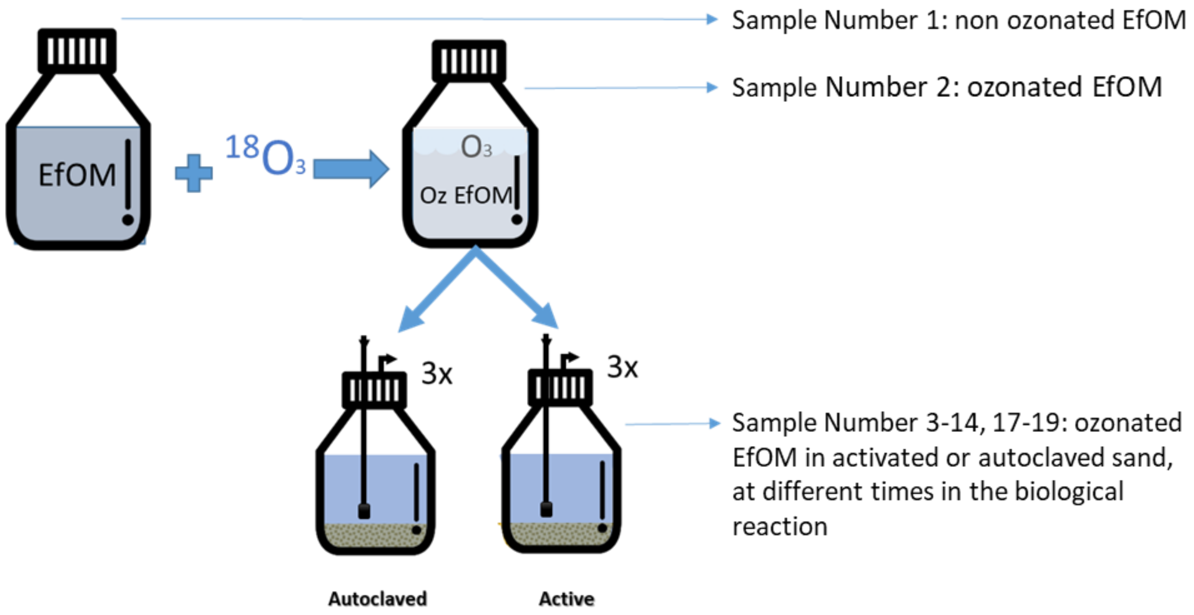

SI Figure 1: Visual Scheme of all samples included in the study. In the sample description, t0 means samples taken at the start of the experiment, t3 means samples taken after 3 days, and t28 means samples taken after 28 days.

SI Table 1: *Set up of active and autoclaved sand bottles.*

Bottle Number	Amount of Sand Added (g)	Amount of ozonated effluent added (mL)
Activated Sand 1	20.190	80
Activated Sand 2	20.020	80
Activated Sand 3	20.140	80
Autoclaved Sand 1	20.171	80
Autoclaved Sand 2	20.006	80
Autoclaved Sand 3	20.171	80

SI Table 2: *List of all samples measured with LC-FT-ICR MS and the aggregate values of each sample. All assigned peaks include multiple assignments (only counted once). Intensity Sum includes all the assigned peak intensities added up for all segments in the chromatogram. In the sample description, t0 means samples taken at the start of the experiment, t3 means samples taken after 3 days, and t28 means samples taken after 28 days.*

Sample Description	Sample #	All Peaks Assigned	Intensity Sum	Mean m/z	Mean H/C	Mean O/C
nOz EfOM	1	71758	2.57E+09	373.0512	1.47	0.48
Oz EfOM	2	67621	1.95E+09	369.7732	1.50	0.51
Batch 1 t0	3	64676	1.89E+09	370.3330	1.48	0.51
Batch 2 t0	4	62757	1.87E+09	366.6388	1.47	0.51
Batch 3 t0	5	66818	1.96E+09	368.3644	1.50	0.50
Autoclave 1 t0	6	114800	3.08E+09	415.7394	1.51	0.44
Autoclave 2 t0	7	115775	3.20E+09	411.1626	1.51	0.44
Autoclave 3 t0	8	127986	3.98E+09	427.5835	1.50	0.43
Batch 1 t3	9	53149	1.04E+09	373.1998	1.45	0.48
Batch 2 t3	10	54081	1.10E+09	365.2685	1.45	0.49
Batch 3 t3	11	55094	1.05E+09	365.4027	1.45	0.49
Autoclave 1 t3	12	63182	1.33E+09	367.2990	1.48	0.50
Autoclave 2 t3	13	90669	2.12E+09	380.6802	1.47	0.49
Autoclave 3 t3	14	106073	2.50E+09	383.2328	1.48	0.49
nOz MQW (TUM)	15	14911	3.61E+08	372.1066	1.45	0.40
Oz MQW (TUM)	16	14766	3.58E+08	370.5362	1.45	0.40
Batch 1 t28	17	52802	1.11E+09	370.3678	1.43	0.47
Batch 2 t28	18	53925	1.13E+09	372.1218	1.44	0.47
Batch 3 t28	19	53493	1.12E+09	371.4986	1.44	0.47
Autoclave 1 t28	20	94869	2.24E+09	376.4056	1.44	0.48
Autoclave 2 t28	21	94595	2.26E+09	380.6797	1.44	0.49
Autoclave 3 t28	22	98463	2.41E+09	381.9277	1.43	0.47
MQW Blank (QC)	23	13978	5.56E+08	355.9062	1.46	0.41

3. Total Ion Chromatograms

Below are the TICs for all samples measured via LC-FT-ICR MS

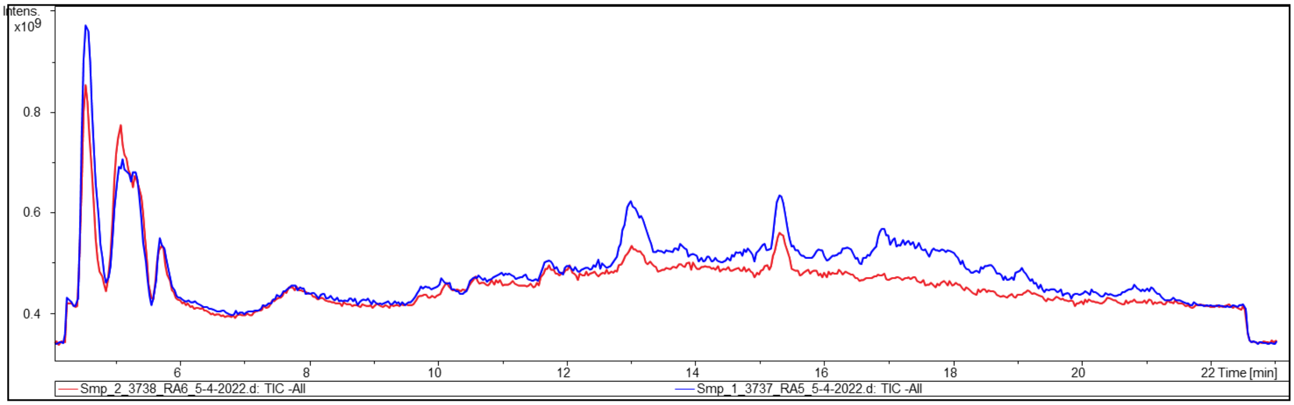

SI Figure 2: *Ozonated (red) and non-ozonated (blue) EfOM, not mixed with activated sand.*

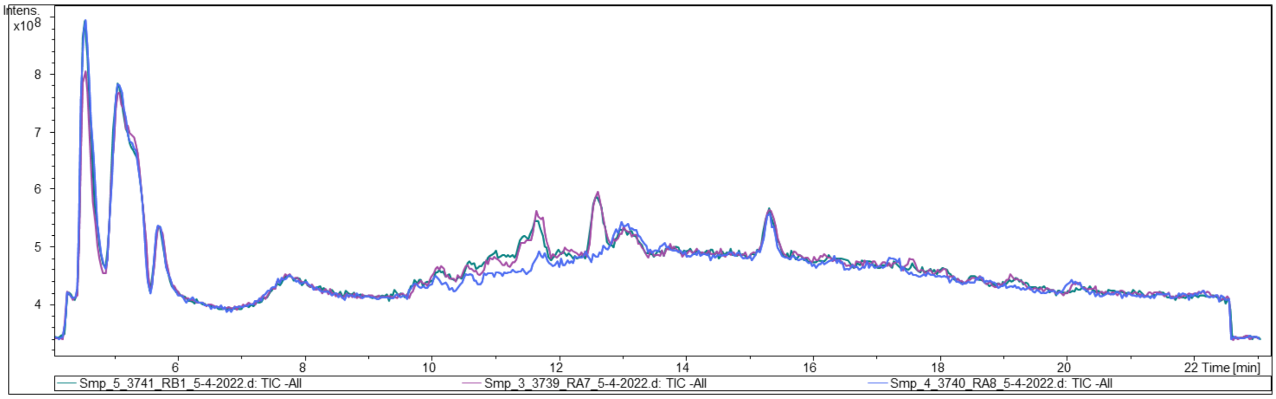

SI Figure 3*: Activated sand bottles 1 (purple), 2 (blue), and 3 (green) immediately after mixing (time=0).*

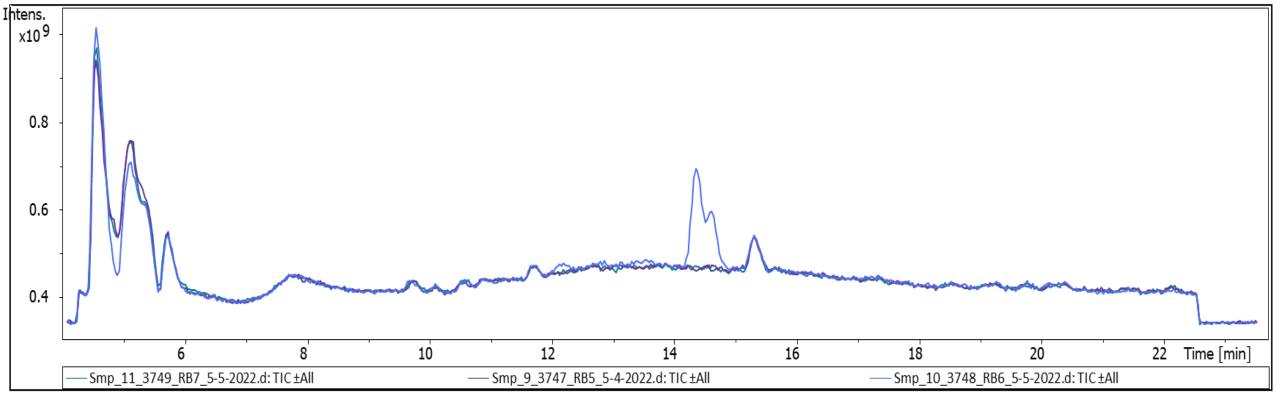

SI Figure 4: *Activated sand bottles 1 (purple), 2 (blue), and 3 (green) after 3 days of shaking in the dark (t=3).*

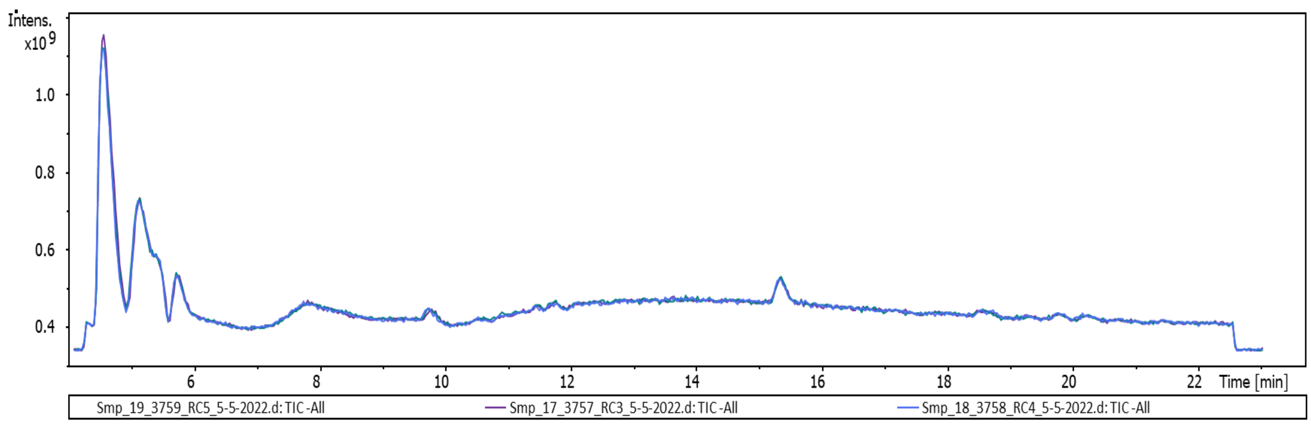

SI Figure 5: *Activated sand bottles 1 (purple), 2 (blue), and 3 (green) after 28 days of shaking in the dark (t=28).*

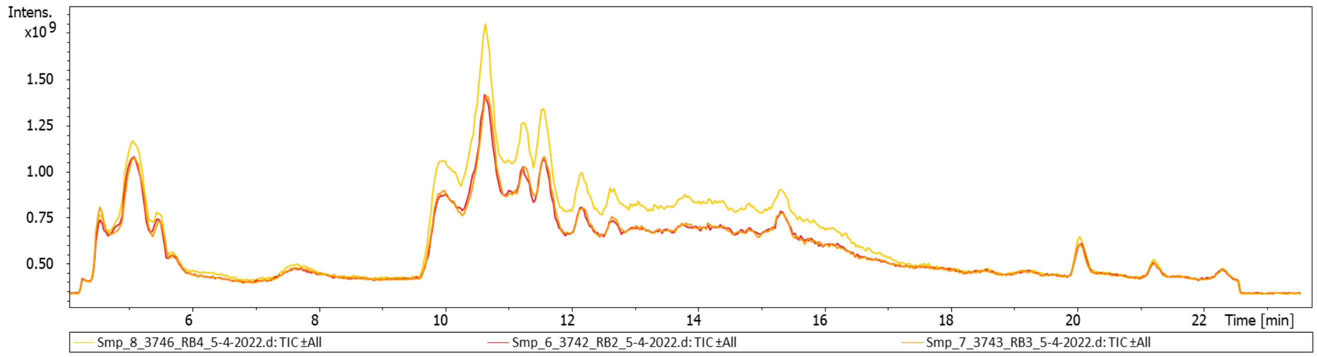

SI Figure 6: *Autoclaved (inactive) sand bottles 1 (red), 2 (orange), and 3 (yellow) immediately after mixing (time=0).*

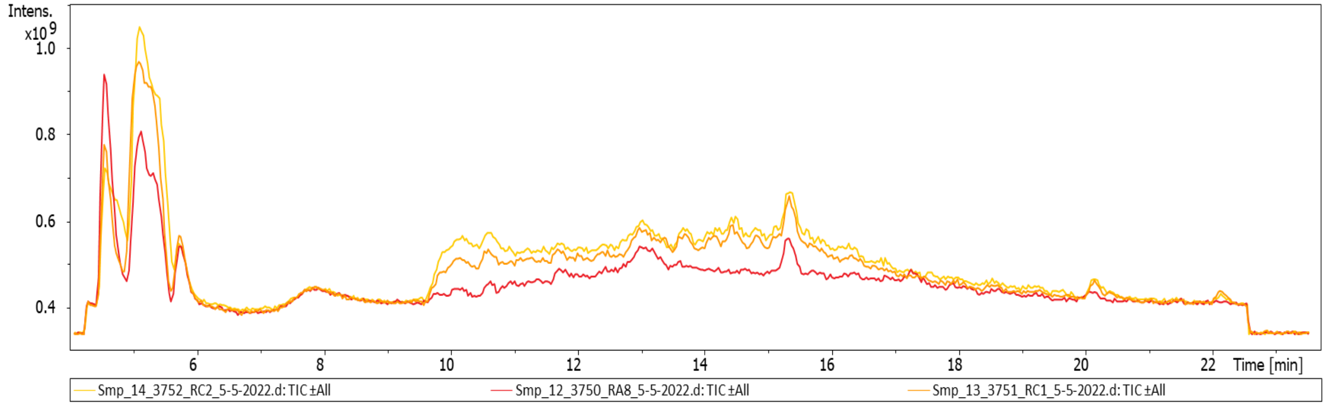

SI Figure 7: *Autoclaved (inactive) sand bottles 1 (red), 2 (orange), and 3 (yellow) after 3 days of shaking in the dark (t=3).*

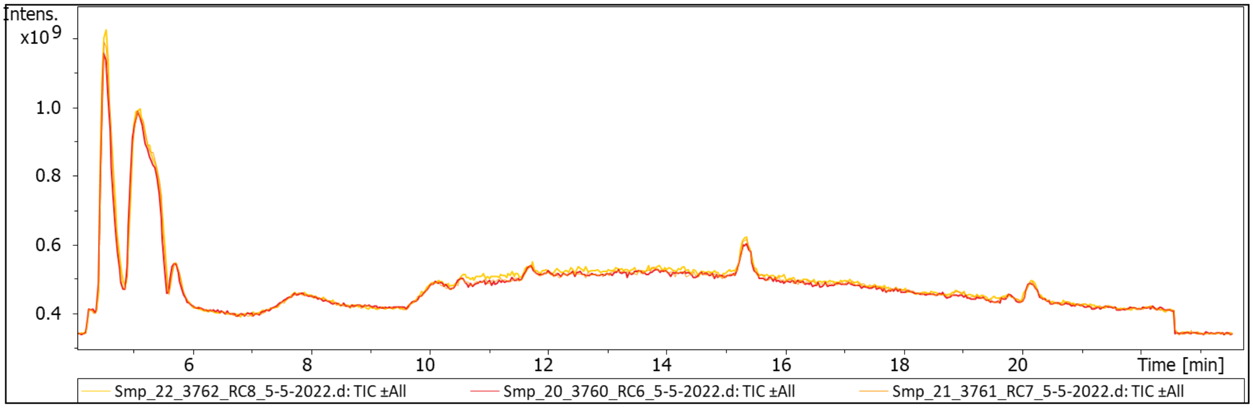

SI Figure 8: *Autoclaved (inactive) sand bottles 1 (red), 2 (orange), and 3 (yellow) after 28 days of shaking in the dark (t=28).*

4. Internal Standard Normalization

To understand which normalization method is more appropriate for the LC-FT-ICR MS analysis of biodegradation data, the performance of internal standard normalization with post-column infusion of labeled naproxen (ISN) according to Rodrigues Matos et al. 2024 was compared to two standard post-hoc normalization methods - when the analytes signals are adjusted to a common denominator by different approaches: the sum of the intensity of all DOM peaks (SUM); most intense (base peak – BPK). The repeatability of five model compounds (MCs used for quality control in LC-FT-ICR MS, cf. Patriarca et al. 2018, Han et al., 2021) 2-(4-(2,2-dicarboxy-ethyl)-2,5-dimethoxy-benzyl)-malonic acid (tR 14.25 min), fraxin (tR 16.04 min), isoferulic acid 3-O-ß-D-glucuronide (tR 15.06 min), leu-enkephalin (tR 16.13 min), and vanillic acid (tR 15.34 min) along a measurement sequence was assessed as the coefficient of variation for the three normalization methods (ISN, BPK and TIC). The MCs were spiked in different SRFA concentration and injected as triplicates. The linearity of the method was assessed with the regression analysis of the individual normalized DOM peak intensities against SRFA carbon concentration.

Independent of SRFA carbon concentration, ISN normalized MCs showed, on average, 10 times less variability than BPK and TIC normalized MCs (SI Figure 9). The mean CV of ISN normalized intensities for all MCs was 12% while BPK and TIC 112 % and 116%, respectively. TIC assume that the number of ions in the ICR cell might be roughly related with the sample carbon concentration. As a consequence, the total ion count is constant, which is appropriate if the samples were normalized to carbon concentration prior to data acquisition. However, when another analytical dimension is used (e.g. online LC), the sample composition changes across the chromatography run and, consequently, the TIC cannot be considered constant anymore, introducing higher variability and making inter-sample comparisons inaccurate. BPK presented higher variability for lower SRFA concentrations (mean of 96 % at 2 and 10% at 10 mg L-1 C). The base peak of lower carbon concentrations is inherently more prone to variation when compared to higher concentrations and is much more dependent on matrix effects. Neither conventional normalization methods (developed for DI-FT-ICR MS with harmonized carbon concentration prior injections) are appropriate for samples measured with variable DOC concentrations and LC-FT-ICR MS.

The results from the MCs show that in order to accurately reflect a specific compounds' concentration, neither TIC nor BPK will work, if the bulk of the DOM/EfOM changes (e.g. during biodegradation). Since in this study we explicitly want to follow the degradation of OPs along the biodegradation experiment, only an independent normalization method is feasible. ISN can thus be used to track the concentration changes of OPs during the incubation.

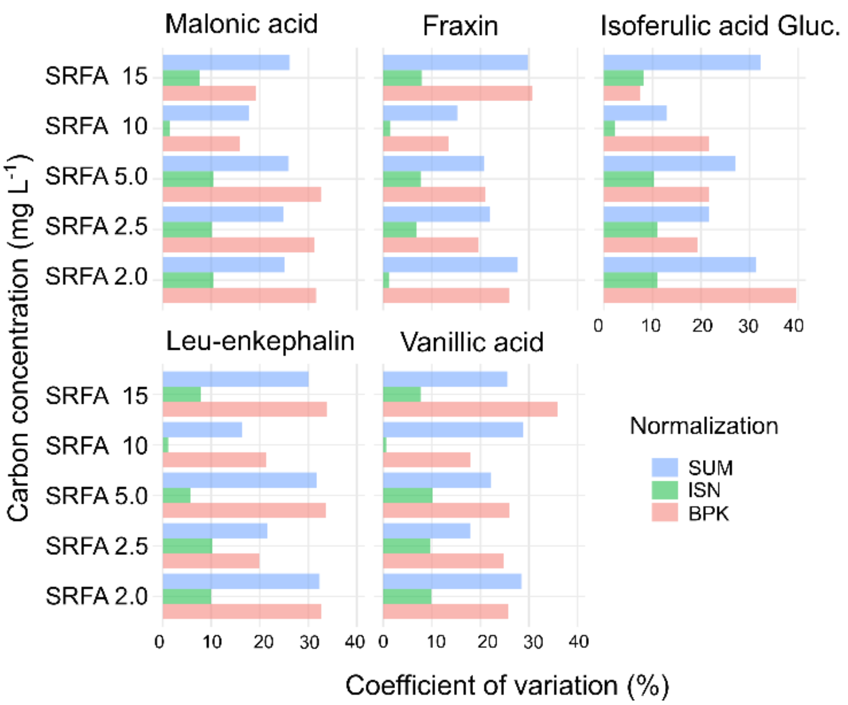

SI Figure 9: Repeatability of the MCs expressed as the coefficient of variation (CV) of mass peak intensity across triplicate injections. MCs were spiked in different SRFA carbon concentrations: 2.0, 2.5, 5.0, 10 and 15 mg/L C. The CVs were calculated for total ion count (SUM, blue), internal standard (ISN, green), and base peak (BPK, pink) normalization.

To understand if the normalized intensity correlates linearly with increased in compound concentration, a linear regression analysis was performed for DOM MFs detected in SRFA with different carbon concentrations. A linear range between 2 and 15 mg C/L was established for SRFA (p-values < 0.001). Within the tested range, the linear regression of ISN values versus sample carbon concentration was significant (with correlation coefficient > 0.9, α = 0.005) for 98% of DOM MFs, which is better than both BPK and TIC (10% and 1% of DOM MFs, respectively, SI Figure 10). TIC is inherently unable to generate correlations between relative intensities and concentrations between samples while DOM BPK was highly influenced by lower carbon concentrations. Previous study performed with LC-FT-ICR MS for ozonation byproducts detection also found that BPK and SUM change the significance of peaks not allowing the comparison between segments and samples (Jennings et al., 2022). This may happen because, during the online LC run, the number of ions in the cell shifts with time (RT), consequently, the sum of intensity of all peaks detected also changes across the RTs. For higher RTs the total ion count is expected to be lower than earlier RTs, since the overall intensity of the later RTs detected peaks are lower. The post-column infusion of internal standard overcomes these limitations, offering more robust comparisons of peak intensity between samples and within samples when measured with LC-FT-ICR MS.

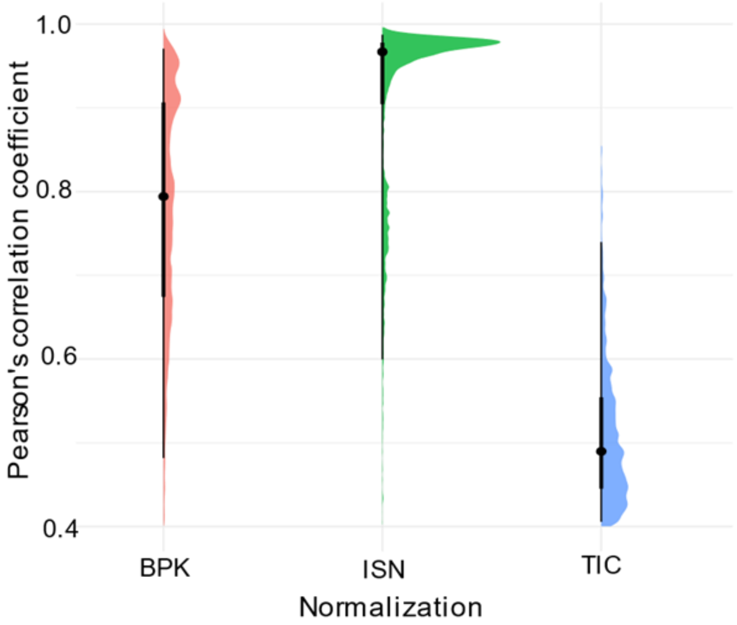

SI Figure 10: *Distribution of Pearson's correlation coefficient for individual DOM MFs, grouped and colored by normalization method (across concentration range 2 – 15 mg L-1). The correlations were calculated independently for each segment (RT 11 - 18 min) and then combined.*

5. $^{18}O/^{16}O$ Data Filtering

Approximately 55% of the oxygen in the ozone generator used during this experiment was labeled (^{18}O), leading to an expected ratio of $^{18}O/^{16}O$ in the labeled OBPs. The following shows the expected ratios based on the relative abundance of each isotope and the number of oxygens added to the molecular formula. Below, n= the number of ^{18}O atoms added to the molecule.

***SI Table 3:** Expected Ratios of $^{18}O/^{16}O$ with 55% abundance of ^{18}O*

	^{16}O	^{18}O	$^{18}O_2$	$^{18}O_3$
n=0	1			
n=1	1	1.2		
n=2	1	2.4	1.5	
n=3	1	3.7	4.5	1.8

Adding an extra 10% abundance to broaden the window of detection, the following limits were used in the data filter. Since no more than n=3 ^{18}O atoms were detected in the dataset, the maximum ratio is 10.3 (highlighted in yellow). Any ^{18}O molecular formula with an intensity higher than 10.3 times that of its ^{16}O isotopologue was removed from the dataset as a false assignment.

***SI Table 4:** The limit of $^{18}O/^{16}O$ ratios, with 65% ^{18}O abundance maximum*

	^{16}O	^{18}O	$^{18}O_2$	$^{18}O_3$
n=0	1			
n=1	1	1.9		
n=2	1	3.7	3.7	
n=3	1	5.5	10.3	6.4

All of the samples were used to generate a final list of validated formulas with and without ^{18}O. The following scheme outlines each data filtering step and which samples were used at which point to generate the final list.

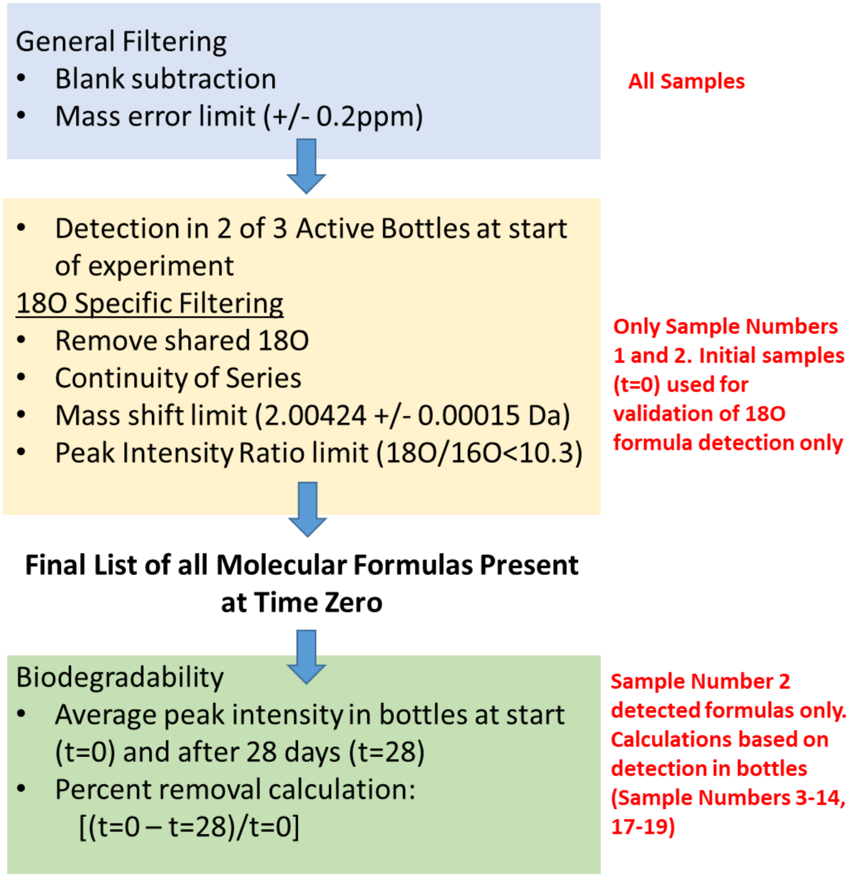

SI Figure 11: *Data processing workflow outline.*

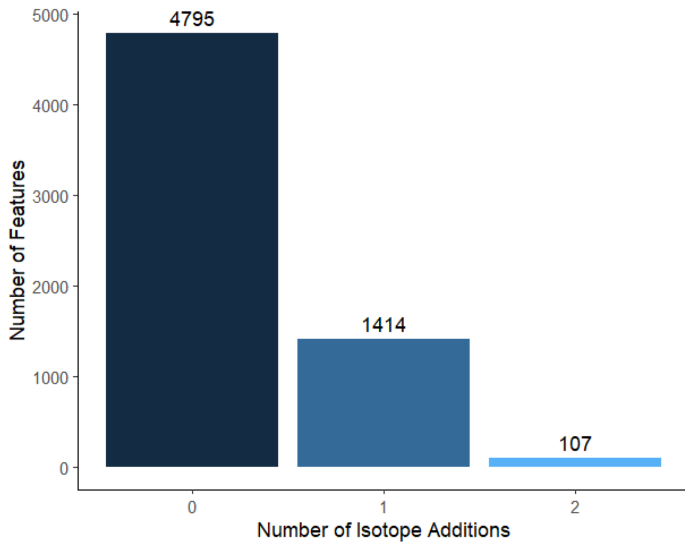

SI Figure 12: *After filtering, the number of ^{18}O isotopes added to all OPs detected. 1414 OPs were detected with one ^{18}O label while 107 were detected with two. Dark blue represents features without any label, while lighter blue represents formulas with the ^{18}O present.*

A filtering limit of O/C ratio was included, limiting the maximum O/C ratio to 1.2. The vast majority of DOM features detected are captured within this limit, with only 117 features found between 1.1-1.2.

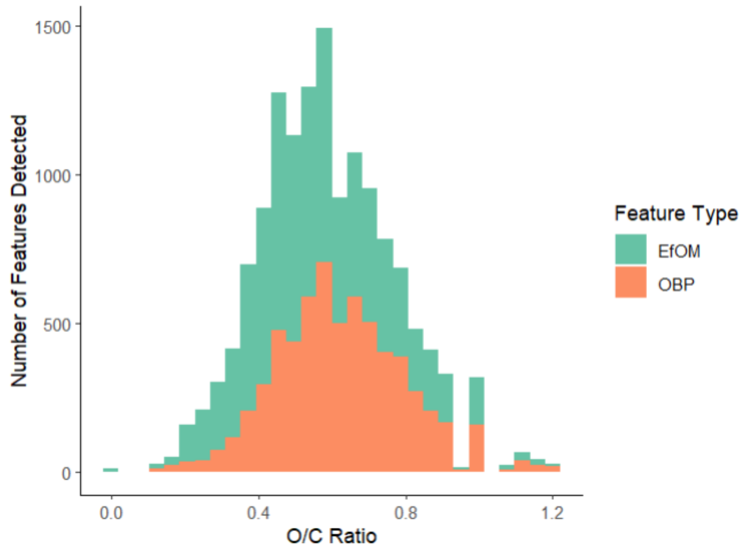

SI Figure 13: *The O/C ratio of all features detected in ozonated EfOM with LC-FT-ICR MS. All OPs are in orange, and the rest of EfOM is in green.*

6. Bulk parameter

SI Table 5: Change in pH, dissolved oxygen, and DOC concentration in bottles over time.

	t= 0 days			t= 3 days			t= 28 days		
**Bottle Number**	**pH**	**Dissolved Oxygen (mg/L)**	**DOC (mg/L)**	**pH**	**Dissolved Oxygen (mg/L)**	**DOC (mg/L)**	**pH**	**Dissolved Oxygen (mg/L)**	**DOC (mg/L)**
Activated Sand 1	7.87	8.32	7.2	8.53	8.57	4.1	8.50	7.65	6.2
Activated Sand 2	7.85	9.48	6.2	8.55	8.44	4.5	8.50	7.70	6.5
Activated Sand 3	7.87	9.25	10.3	8.55	8.40	5.7	8.45	7.71	7.3
Autoclaved Sand 1	7.91	8.53	19.2	8.55	8.60	16.4	8.54	7.74	15.0
Autoclaved Sand 2	7.94	9.28	17.7	8.58	8.50	15.4	8.56	7.62	15.6
Autoclaved Sand 3	7.92	8.39	25.1	8.57	8.37	16.6	8.54	7.66	17.2

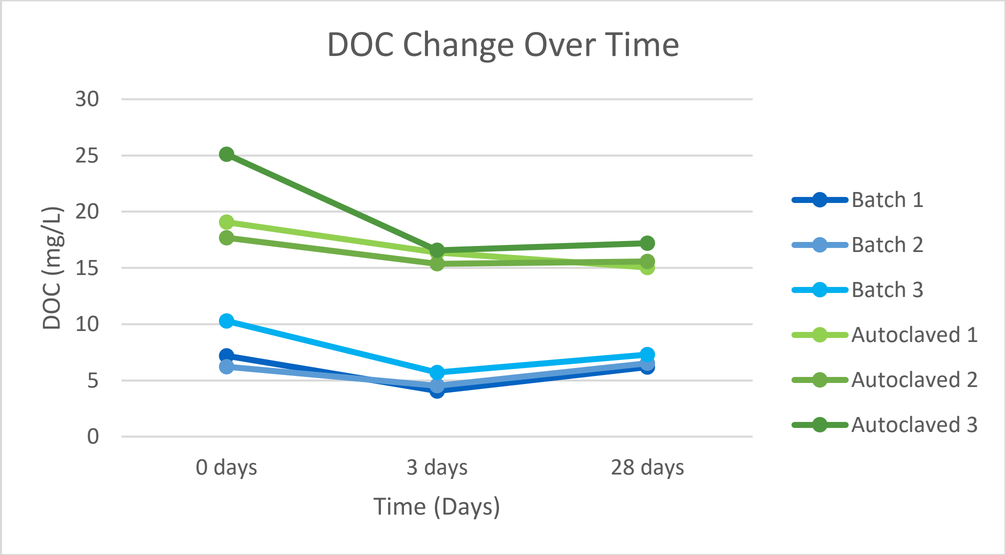

SI Figure 14: Change in DOC concentration over time in each bottle. Blue: activated sand, green: autoclaved sand.

7. Reactivity with Ozone and Biological Treatment

Effluent was first classified based on the reactivity with ozone. In the below tables, EfOM Type refers to whether the molecular formula was non-reactive (i.e., no intensity change after ozonation), depleted, or produced during the ozonation process.

SI Table 6: Formula class distribution based on reactivity with ozone.

EfOM Type	Formula Class	Number of Formulas
Non-reactive	CHNO	1572
	CHO	868
	CHOS	450
	CHNOS	76
Depleted	CHNO	2063
	CHO	1959
	CHOS	683
	CHNOS	106
OP	CHNO	2935
	CHO	2225
	CHOS	934
	CHNOS	220

SI Table 7: Distribution of formula classes in EfOM MF with different reactivity towards ozone.

	CHO	CHNO	CHOS	CHNOS
Depleted	39%	31%	33%	26%
OP	44%	45%	45%	55%
Non-reactive	17%	24%	22%	28%

Biodegradability classification was based on the percent intensity change between the start of the experiment (time=0) and after 3 days, or after 28 days in the bioreactors. Averages were calculated using three bottles per treatment (activated and autoclaved sand).

We consider the initial decrease in DOC concentration in the abiotic control by removing all compounds that show a larger decrease in the abiotic controls from the active sand incubations. We distinguish compounds initially degraded after 3 days that remain depleted after 28 days as readily biodegradable and compounds that only show degradation after 28 says as biodegradable. The increase in DOC concentration towards the end of the active sand incubation implies a release of DOC from biomass or the particulate matrix over the course of the experiment. If a compound was biologically produced or released from the active sand during incubation, if would be classified as bio-produced. Consequently, only compounds with a near-zero net intensity change are classified as recalcitrant. However, as compared to traditional DI analysis, the introduction of ^{18}O labeling for OPs and use of LC-FT-ICR MS

(allowing isomeric differentiation on the polarity level) substantially increases the confidence that a recalcitrant OP (with net-zero change in intensity) indeed was neither degraded nor produced by biological activity.

SI Table 8: Thresholds for biodegradability classification

Biodegradability Classification	Percent Intensity Change after 3 days	Percent Intensity Change after 28 days
Bio-produced	--	≥30%
Recalcitrant	--	>-30%, <30%
Biodegradable	--	≤ -30%
Readily biodegradable	≤ -30%	≤ -30%
Fully removed	--	Below Detection§

§ "Below detection" refers to MF not detected or only found in one out of the three bottle replicates after 3 or 28 days.

SI Table 9: Total number of features found in ozonated EfOM based on removal classification (abiotic removed not included) and the average retention time for each removal class.

	Number of Features in EfOM	Average Retention Time (min) for All	Average Retention Time (min) for OBPs only
Fully removed	3191	12.2	8.2
Readily biodegradable	3171	11.5	8.4
Biodegradable	1671	11.8	7.6
Recalcitrant	1476	10.9	7.4
Bio-produced	2103	11.6	8.2

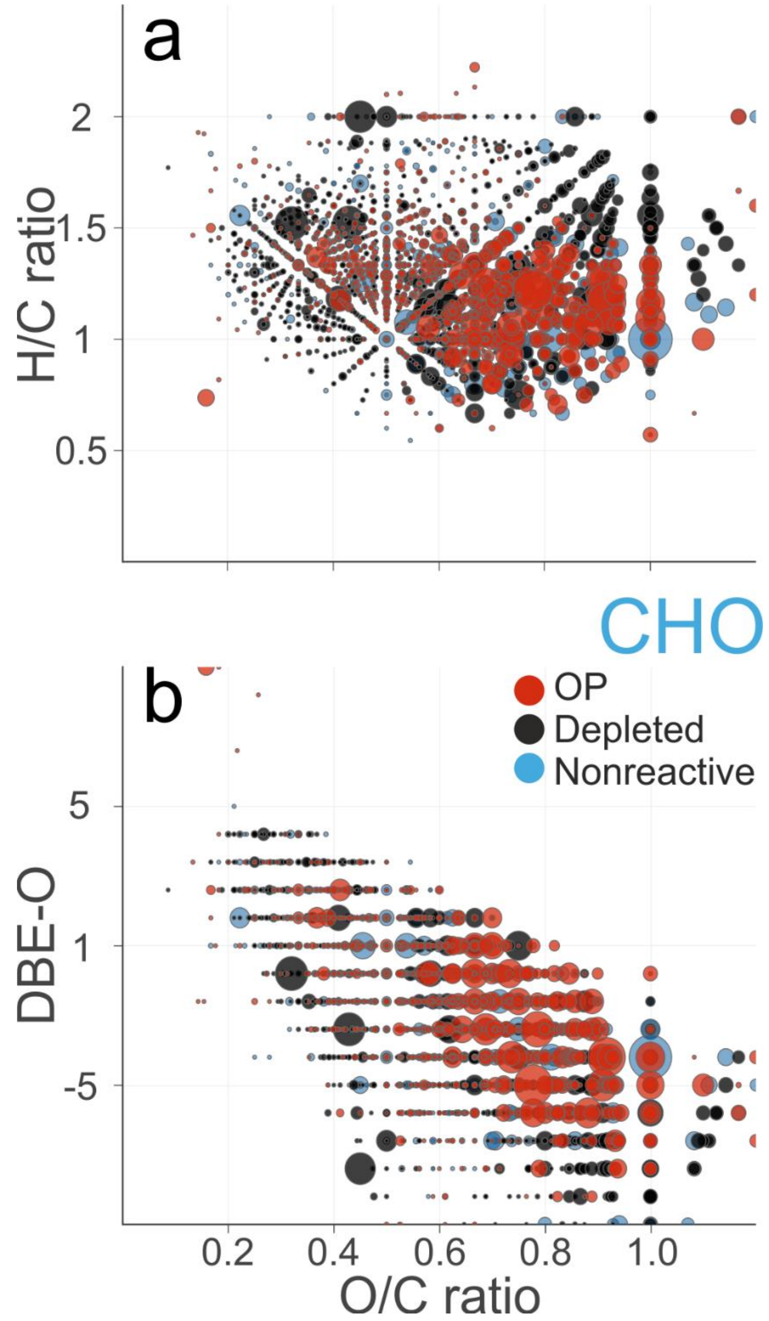

SI Figure 15: *Molecular H/C vs O/C (a) and DBE-O vs O/C (b) values of FT-ICR MS derived molecular formulas and their reactivity classification with ozone (ozonation products, OP, depleted and non-reactive EfOM). Only CHO-containing MF are shown.*

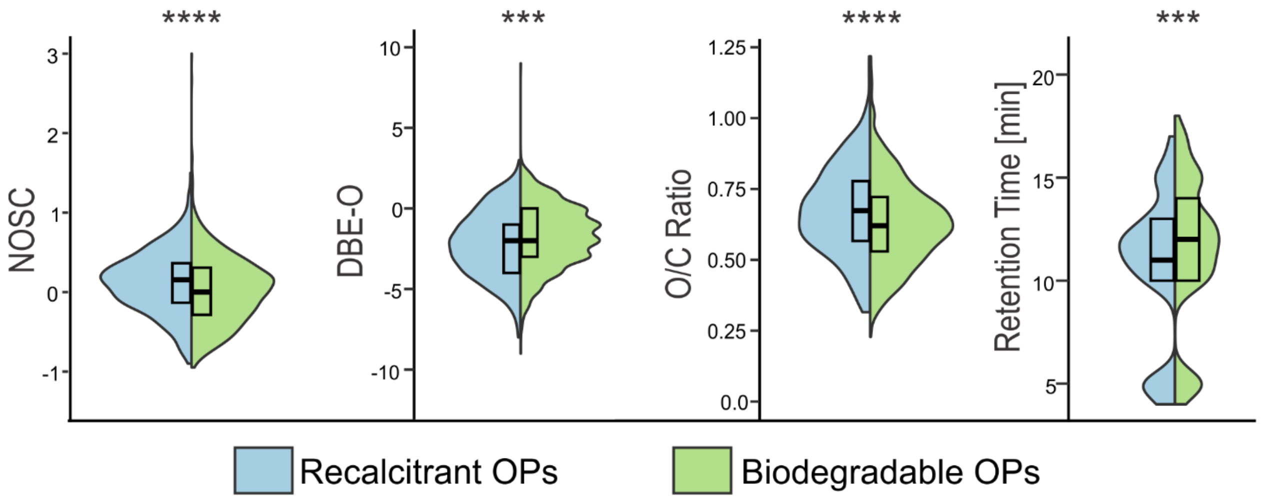

SI Figure 16: *Comparison of biodegradable OPs (including readily biodegradable and biodegradable in one group) (green, n = 1015) and recalcitrant OPs (blue, n = 317) by their molecular descriptors NOSC, O/C, DBE-O, and their retention time in LC-FT-ICR MS, showing only labeled OPs. Half violins are scaled based on the number of detected MFs in each group. The significance levels of differences in mean values is indicated with ***: p-value < 0.001 and ****: p-value < 0.0001.*

8. Correlation between Molecular Descriptors and Biodegradability

SI Table 10: *Multivariable linear model coefficients correlating biodegradability with H/C, NOSC, DBE-O, retention time, and m/z for all recalcitrant, biodegradable, readily biodegradable, and fully removed MFs. The R^2 of the model is 0.064, residual standard error is 0.97, and root mean squared error is 1.0, all based on standardized variables.*

	Regression Coefficient	Std Error	t statistic	p-value	Variance Inflation Factor
Intercept	0.003	0.011	0.25	0.804	--
H/C	0.360	0.018	20.38	< 0.001	2.5
NOSC	0.160	0.017	9.02	< 0.001	2.4
DBE-O	0.200	0.018	11.27	< 0.001	2.6
Retention Time	0.082	0.015	5.30	< 0.001	1.9
m/z	0.130	0.014	9.66	< 0.001	1.5

Notably, the large variability and scatter (see SI Figures 17-21) within the data makes using molecular descriptors derived from MF inaccurate when attempting to predict biodegradability. The predicted vs actual values (see SI Figure 22) demonstrate how poorly the multivariable model can predict removal. The correlation coefficient between molecular descriptors and relative intensity changes after biological treatment is near zero for H/C, O/C, DBE-O, NOSC, and molecular weight, meaning none of these parameters are strong predictors of biodegradability even if the trends are significant. Models to predict biodegradability, e.g. via quantitative structure biodegradability relationships (QSBRs), usually rely on structure specific information for predictions (Cheng et al., 2012; Pizzo et al., 2016; Raymond et al., 2001). Recently, machine learning has been used to find that chemical substructures, such as functional groups, are the best determinant of biodegradability, information that is not available from MS1 data on the ozonated EfOM in this study (Huang and Zhang, 2022).

All linear correlations calculated by Pearson Correlation, including all formulas classified as recalcitrant, biodegradable, and readily biodegradable. Percent removal is how much the signal intensity decreases after 28 days in the bioreactors. Color based on retention time (RT) in minutes.

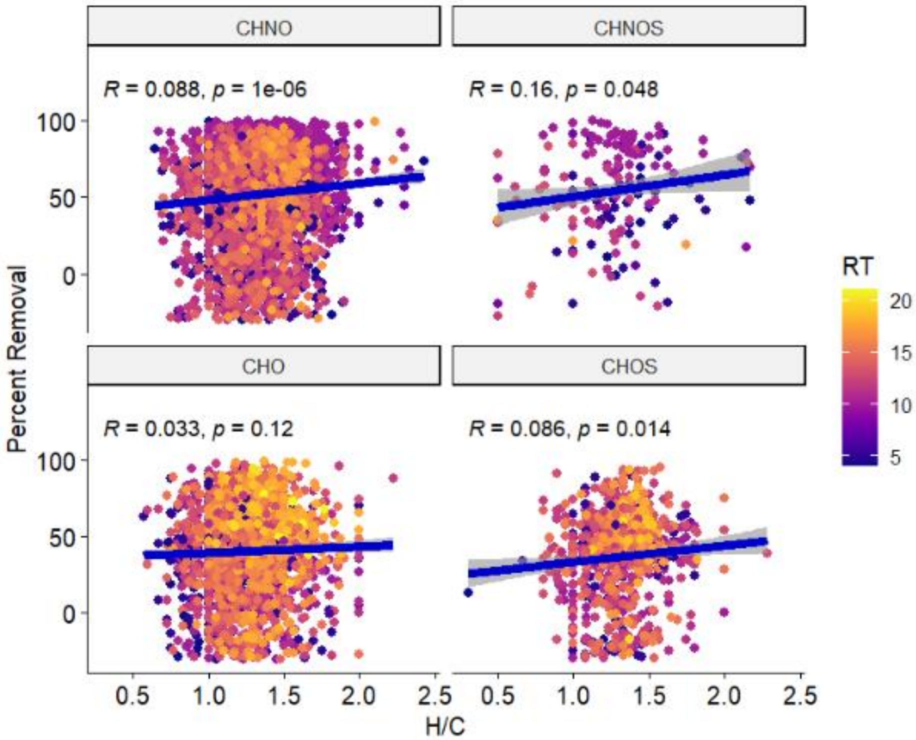

SI Figure 17: *Linear correlation between percent removal and H/C ratio.*

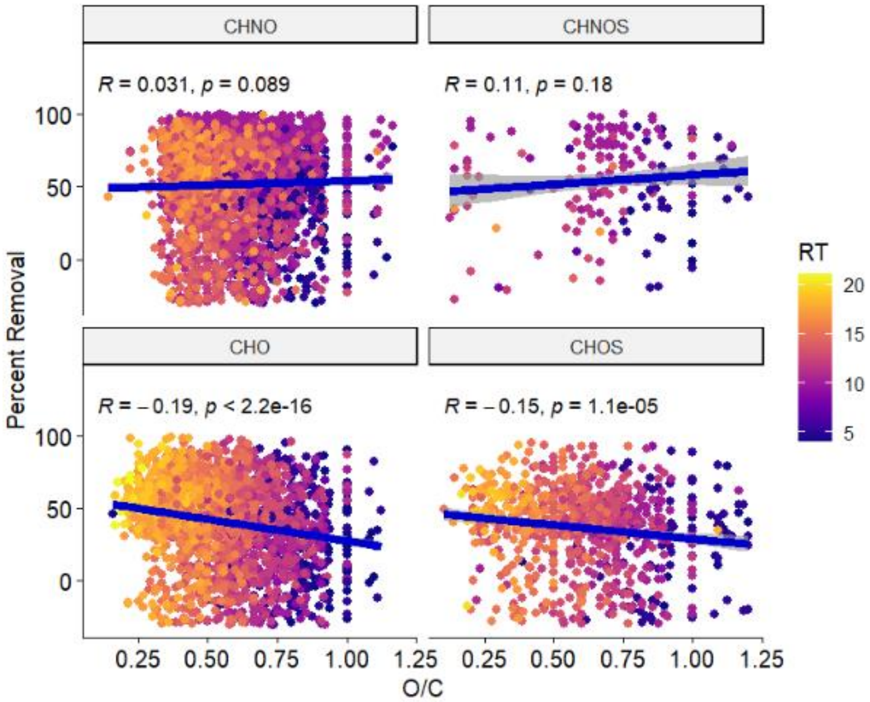

SI Figure 18: *Linear correlation between percent removal and O/C ratio.*

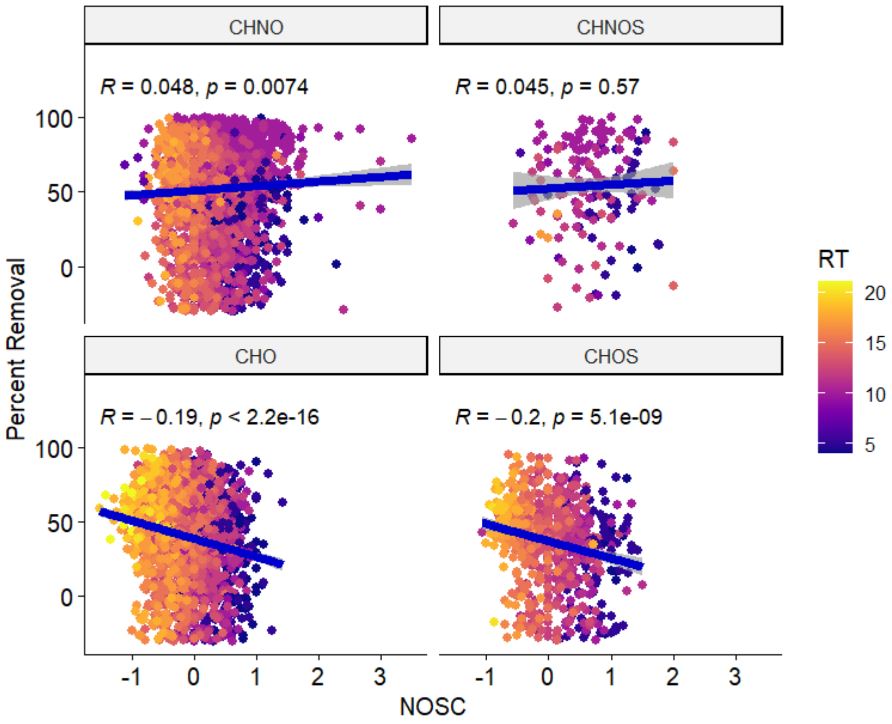

SI Figure 19: *Linear correlation between percent removal and formula NOSC.*

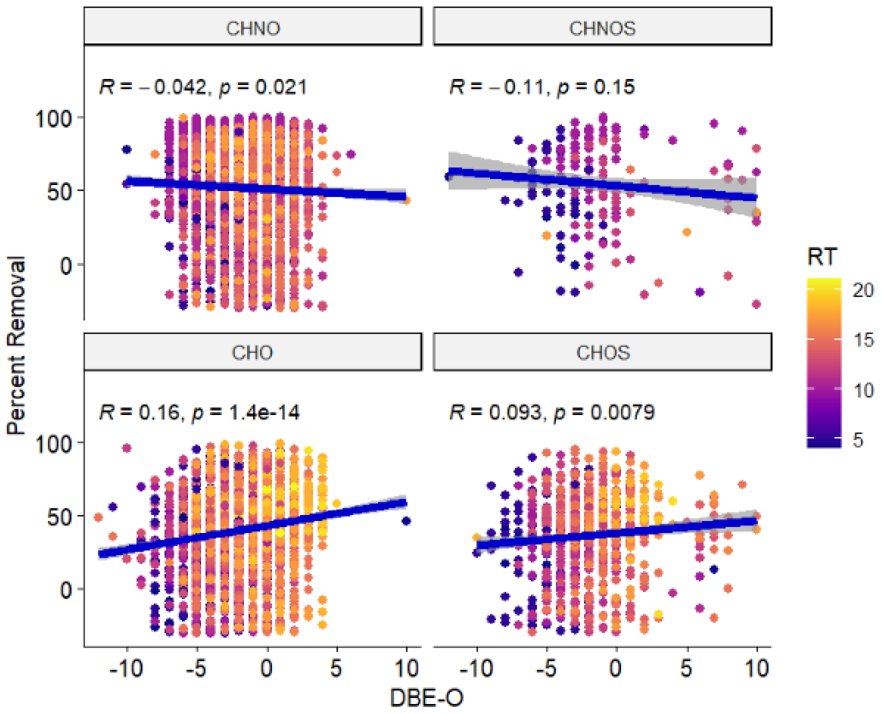

SI Figure 20: *Linear correlation between percent removal and formula DBE-O.*

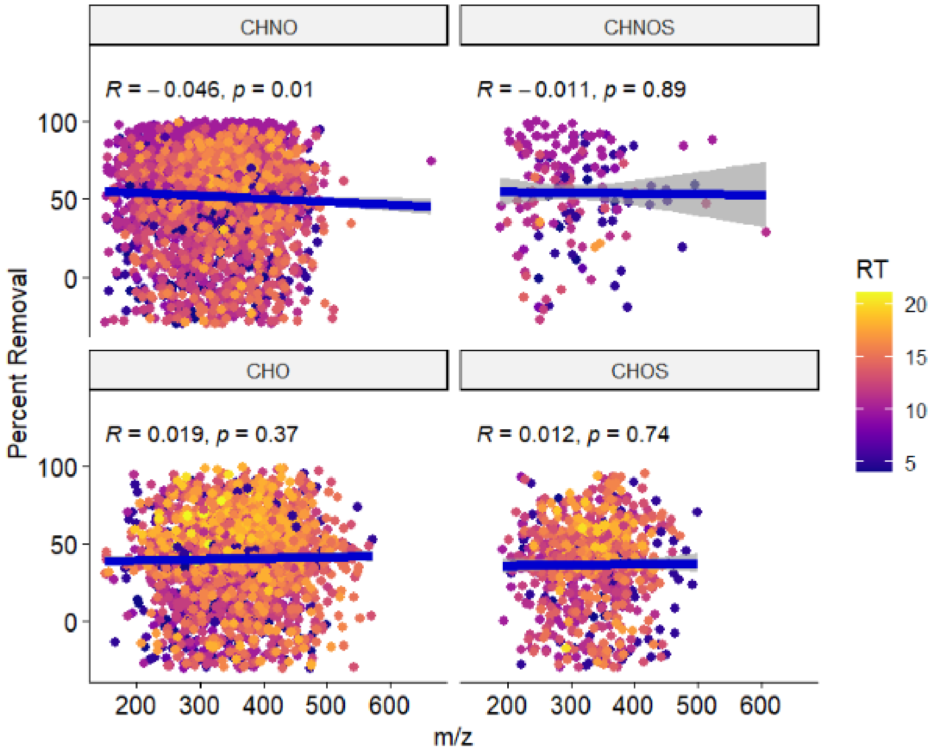

SI Figure 21: Linear correlation between percent removal and molecular weight.

The following plots were generated from the multivariable model after all variables were standardized. The fit of the actual vs predicted removal (SI Figure 22) and the plot of the residuals (SI Figure 23) show how poorly the model is able to predict the removal of compounds in our sample.

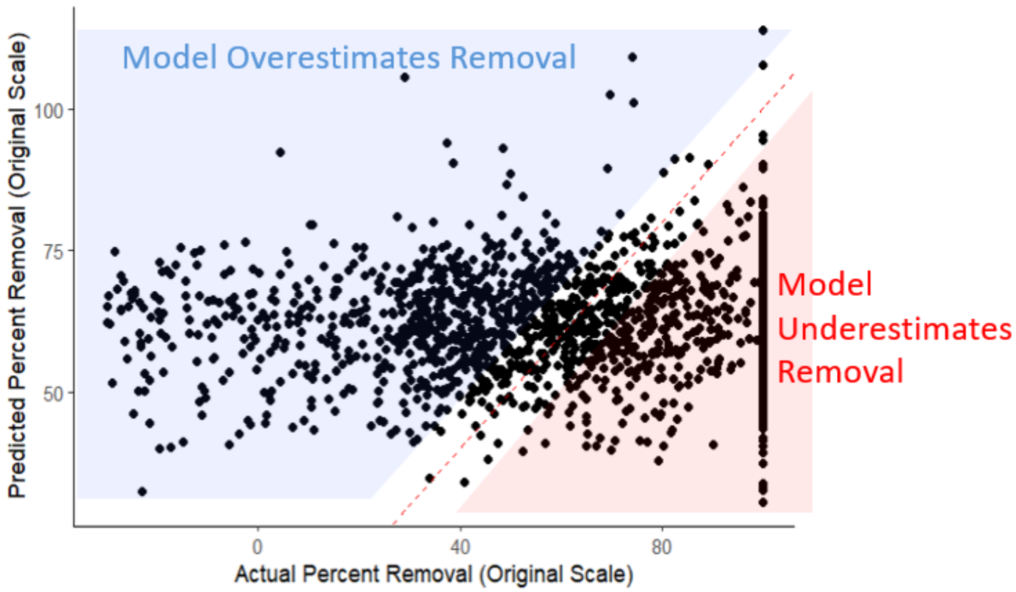

SI Figure 22: The fit of the predicted removal and actual removal of 20% of the EfOM data based on the multivariable linear model. The red dashed line indicates where the model perfectly predicts the removal, while dots below represent cases where the model's predictions underestimate the actual removal (red) and dots above are overestimated by the model (blue).

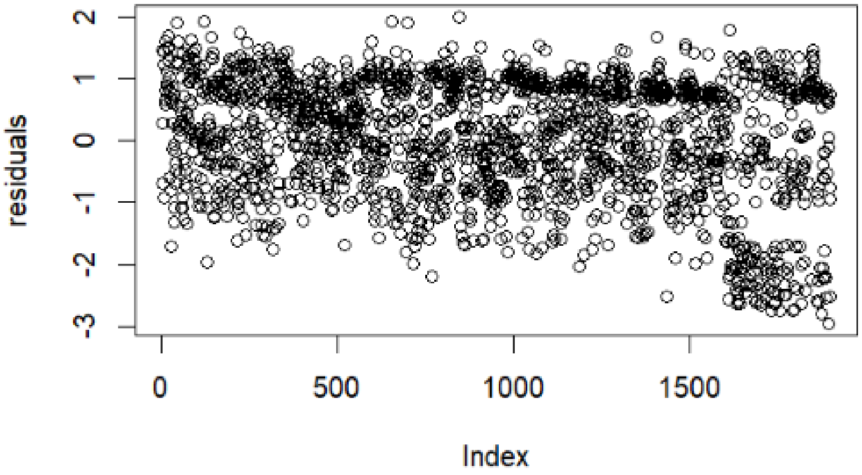

SI Figure 23: Residual plot from the multivariable linear model. Since the data are not evenly distributed around 0, the model does not predict the data well.

9. Isomers in LC-FT-ICR MS

Many MFs are detected multiple times over the course of the chromatographic run. Every detection is presumed to be at least one distinct isomer of the molecular formula detected. SI Figure 24 demonstrates how many isomers are present for each molecular formula based on the number of times each formula is detected in the chromatogram. Most MFs (2,838) are detected more than once, while four MFs are detected a maximum of 11 times. With the current state of data processing, this provides a conservative estimation of the number of isomers present, but does not represent full isomeric composition present in EfOM.

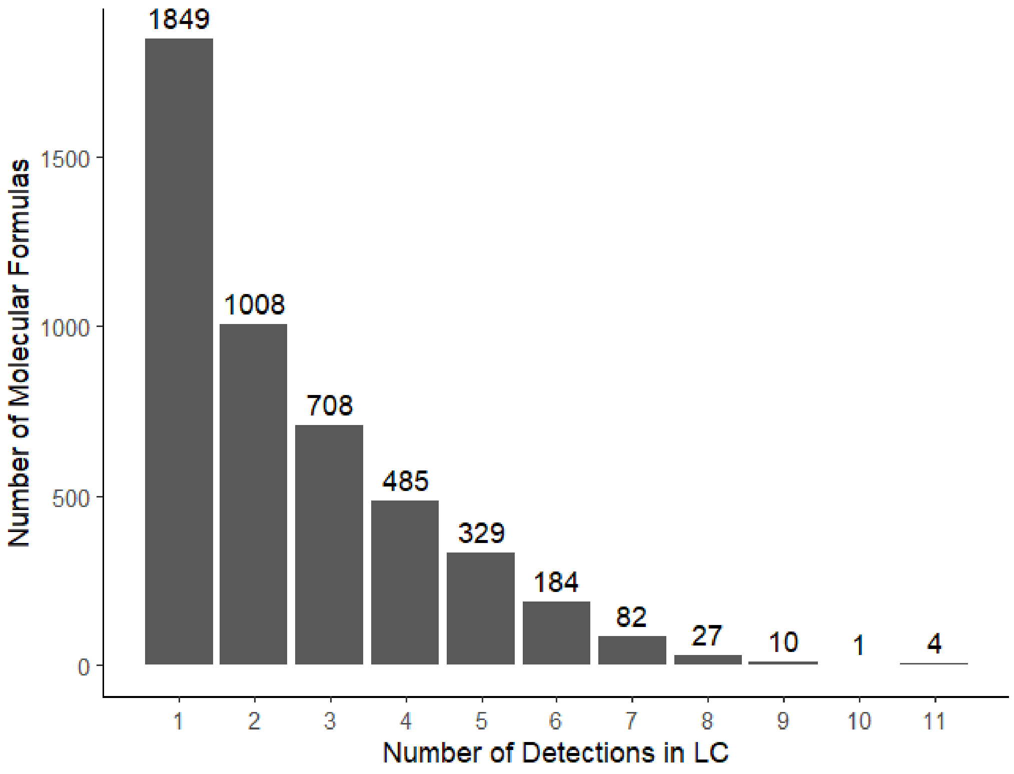

SI Figure 24: *The number of multiple detections over the chromatographic separation for all EfOM molecular formulas. Each detection represents at least one isomer present in the sample.*

Many isomers also have varying levels of biodegradability detected at different retention times. Out of all the MFs with presumed isomers (detected more than once), SI Table 11 shows how many different removal classifications are also detected. SI Figure 25 also shows this data as a distribution of the number of total detections. The more isomers are detected, the higher the likelihood of more classifications being assigned to the same molecular formulas.

SI Table 11: *How many MFs with isomers are detected with different removal classifications (readily biodegradable, biodegradable, recalcitrant, bio-produced, or fully removed).*

Number of Detected Removal Classifications for Isomers	Number of Molecular Formulas
1	413
2	1256
3	837
4	284
5	48

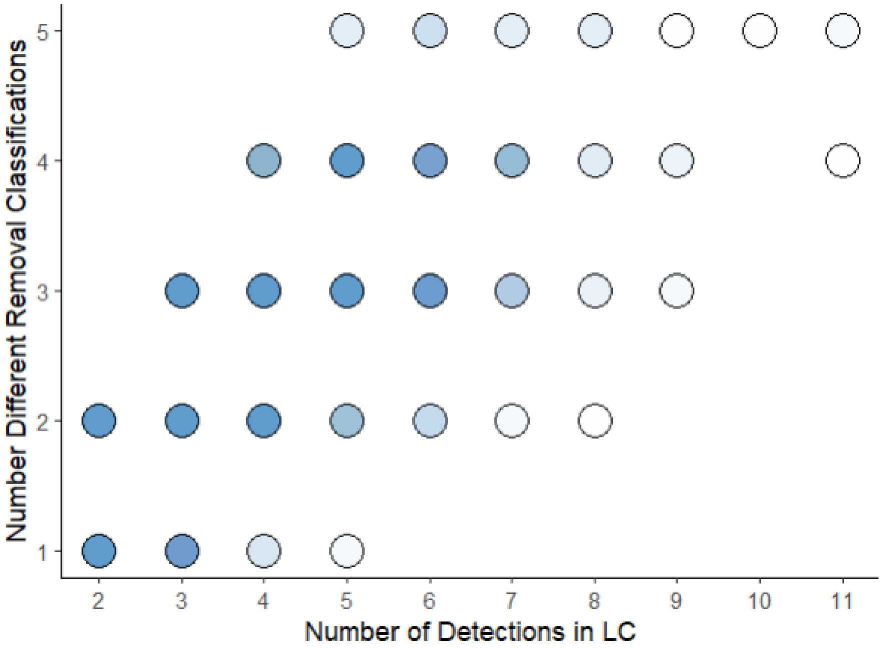

SI Figure 25: *The number of different removal classifications assigned to each MF and the number of times it was detected in LC-FT-ICR MS. The intensity of the colored dots represent the number of MFs with this many detections and removal classifications, with the lightest dots (white) equal to one and the darkest dot (two detections and two different classifications) equal to 698 MFs. The maximum number of distinct classification = 5, relating to the different removal classifications included (readily biodegradable, biodegradable, recalcitrant, bio-produced, or fully removed).*

10. Additional References

Han, L.; Kaesler, J.; Peng, C.; Reemtsma, T.; Lechtenfeld, O. J., Online Counter Gradient LC-FT-ICR-MS Enables Detection of Highly Polar Natural Organic Matter Fractions. *Anal. Chem.* **2021,** *93* (3), 1740-1748. https://doi.org/10.1021/acs.analchem.0c04426

Patriarca, C.; Bergquist, J.; Sjoberg, P. J. R.; Tranvik, L.; Hawkes, J. A., Online HPLC-ESI-HRMS Method for the Analysis and Comparison of Different Dissolved Organic Matter Samples. *Environ. Sci. Technol.* **2018,** *52* (4), 2091-2099. https://doi.org/10.1021/acs.est.7b04508

Rodrigues Matos, R.; Jennings, E. K.; Kaesler, J.; Reemtsma, T.; Koch, B. P.; Lechtenfeld, O. J., Post column infusion of an internal standard into LC-FT-ICR MS enables semi-quantitative comparison of dissolved organic matter in original samples. *Analyst* **2024,** *149* (12), 3468-3478. https://doi.org/10.1039/d4an00119b

Jennings, E.; Kremser, A.; Han, L.; Reemtsma, T.; Lechtenfeld, O. J., Discovery of Polar Ozonation Byproducts via Direct Injection of Effluent Organic Matter with Online LC-FT-ICR-MS. *Environ. Sci. Technol.* **2022,** *56* (3), 1894-1904. https://doi.org/10.1021/acs.est.1c04310

Huang, K.; Zhang, H., Classification and Regression Machine Learning Models for Predicting Aerobic Ready and Inherent Biodegradation of Organic Chemicals in Water. *Environ. Sci. Technol.* **2022,** *56* (17), 12755-12764. https://doi.org/10.1021/acs.est.2c01764

Raymond, J. W.; Rogers, T. N.; Shonnard, D. R.; Kline, A. A., A review of structure-based biodegradation estimation methods. *J. Hazard. Mater.* **2001,** *84* (2-3), 189-215. https://doi.org/10.1016/s0304-3894(01)00207-2

Cheng, F.; Ikenaga, Y.; Zhou, Y.; Yu, Y.; Li, W.; Shen, J.; Du, Z.; Chen, L.; Xu, C.; Liu, G.; Lee, P. W.; Tang, Y., In silico assessment of chemical biodegradability. *J. Chem. Inf. Model.* **2012,** *52* (3), 655-69. https://doi.org/10.1021/ci200622d

Pizzo, F.; Lombardo, A.; Brandt, M.; Manganaro, A.; Benfenati, E., A new integrated in silico strategy for the assessment and prioritization of persistence of chemicals under REACH. *Environ Int* **2016,** *88*, 250-260. https://doi.org/10.1016/j.envint.2015.12.019
